# TGF-β/BMP signaling in skeletal biology: molecular mechanisms, regulatory networks, and therapeutic implications in development, regeneration, and disease

**DOI:** 10.1038/s41413-025-00497-y

**Published:** 2026-01-13

**Authors:** Junguang Liao, Taofen Wu, Qi Zhang, Panpan Shen, Ziyi Huang, Jiaqi Wang, Pengxiang Zhang, Sisi Lin, Jiashun Pi, Nenghua Zhang, Haidong Wang, Guiqian Chen

**Affiliations:** 1 Department of Biopharmaceutics, Zhejiang Provincial Engineering Research Center of New Technologies and Applications for Targeted Therapy of Major Diseases, College of Life Science and Medicine, Zhejiang Sci-Tech University, Hangzhou, China; 2 R&D Department, Ceva Animal Health Technology (Hangzhou) Co. Ltd, Hangzhou, China; 3 Department of Orthopedic Surgery, Jiaxing Traditional Chinese Medicine Hospital, Jiaxing, China; 4 Clinical Center, Jiaxing Traditional Chinese Medicine Hospital, Jiaxing, China

**Keywords:** Physiology, Pathogenesis

## Abstract

The transforming growth factor-β (TGF-β) and bone morphogenetic protein (BMP) signaling pathways are pivotal regulators of cellular processes, playing indispensable roles in embryogenesis, postnatal development, and tissue homeostasis. These pathways are particularly critical within the skeletal system, as they coordinate osteogenesis, chondrogenesis, and bone remodeling through intricate molecular mechanisms. TGF-β/BMP signaling is primarily transduced via canonical Smad-dependent pathways (e.g., ligands, receptors, and intracellular Smads) and the non-canonical Smad-independent (e.g., p38 mitogen-activated protein kinase, MAPK) cascade. Both pathways converge on master transcriptional regulators, including Runx2 and Osterix, and their precise coordination is indispensable for skeletal development, maintenance, and repair. The dysregulation of TGF-β/BMP signaling contributes to a spectrum of skeletal dysplasia and bone pathologies. Advances in molecular genetics, particularly gene-targeting strategies and transgenic mouse models, have deepened our understanding of the spatiotemporal control of TGF-β/BMP signaling in bone and cartilage development. Moreover, emerging research underscores extensive crosstalk between TGF-β/BMP and other critical pathways, such as Wnt/β-catenin, mitogen-activated protein kinase (MAPK), parathyroid hormone (PTH)/PTH-related protein (PTHrP), fibroblast growth factors (FGF), Hedgehog, Notch, insulin-like growth factors (IGF)/insulin-like growth factors receptor (IGFR), Mammalian target of rapamycin (mTOR), and autophagy, forming an integrated regulatory network that ensures skeletal integrity. Our review synthesizes the current knowledge on the molecular components, regulatory mechanisms, and functional integration of TGF-β/BMP signaling in skeletal biology, with an emphasis on its roles in development, regeneration, and disease. By elucidating the molecular underpinnings of TGF-β/BMP pathways and their contextual interactions, we aim to highlight translational opportunities and novel therapeutic strategies for treating skeletal disorders.

## Introduction

The skeletal system serves as the cornerstone of the structural integrity and functional dynamics of the body, safeguarding and facilitating the development and maturation of the brain and other organ systems. Beyond its structural role, it is actively involved in systemic metabolism, endocrine function, tissue remodeling, and calcium homeostasis throughout life. Bone formation occurs through two principal processes: endochondral ossification, which relies on a cartilage template,^1–3^ as observed in long bones and ribs, and intramembranous ossification, in which the bone forms directly from condensed skeletal mesenchymal stem cells (MSCs) without a cartilage intermediate, as observed in the skull vault.^4–6^ The development, growth, and metabolic turnover of bone are meticulously regulated by numerous signaling pathways and molecular cues orchestrated through complex interactions among osteoblasts, osteoclasts, osteocytes, chondrocytes, MSCs, and cranial neural crest cells (CNCs). CNCs are essential for craniofacial morphogenesis, with CNC-derived frontal bones exhibiting superior osteogenic potential.^5,7–13^ Throughout life, the skeleton is vigorously and continuously remodeled. Disruptions to intercellular communication within this network can lead to severe congenital anomalies, skeletal dysplasia,^14–16^ and disorders such as osteoporosis and osteoarthritis (OA).^17–20^ A deeper understanding of the regulatory mechanisms governing skeletal development and maintenance is crucial to address these pathologies and develop targeted therapeutic interventions.

The transforming growth factor-beta (TGF-β) superfamily comprises over 40 ligands, including TGF-βs, bone morphogenetic proteins (BMPs), Nodal, and Activin.^2,11,21,22^ Among these, the TGF-β and BMP signaling pathways are paramount in skeletal development and metabolism. Pathogenic variants in genes associated with TGF-β/BMP signaling or the regulation of their bioavailability are implicated in more than 30 distinct types of skeletal dysplasia,^14–16,23^ underscoring the indispensable roles of these pathways. TGF-β/BMP signaling is initiated at the cell surface through the ligand-induced assembly of heteromeric complexes of type-I and -II serine/threonine kinase receptors.^24,25^ This triggers the transphosphorylation of the type-I receptor,^26^ which then activates receptor-regulated Smad proteins (R-Smads) through C-terminal phosphorylation. Activated R-Smads form complexes with Smad4 (co-Smad), which translocate into the nucleus to modulate transcriptional programs in skeletal cell types, such as osteoblasts, chondrocytes, and MSCs.^27^ This finely tuned signaling cascade is essential for coordinating skeletal development, remodeling, and homeostasis, and its dysregulation can lead to profound skeletal abnormalities, necessitating further research for the development of targeted therapeutic interventions.

In this review, we summarize recent advances in our understanding of the molecular components and regulatory mechanisms of TGF-β/BMP signaling in osteogenesis, bone and cartilage development, skeletal homeostasis, regeneration, and disease. We highlight the intricate gene regulatory networks that integrate TGF-β/BMP signaling with other critical pathways, including mitogen-activated protein kinase (MAPK), parathyroid hormone (PTH)/PTH-related protein (PTHrP), fibroblast growth factors (FGFs), Hedgehog, Notch, insulin-like growth factors (IGFs)/insulin-like growth factors receptor (IGFR), mammalian target of rapamycin (mTOR), and autophagy, within the skeletal system. Elucidating this crosstalk and coordination provides a foundation for designing novel molecules that target TGF-β/BMP signaling. Such advancements hold significant promise for the development of strategic therapies to treat a wide spectrum of bone-related disorders.

## TGF-β signaling components in the skeletal system: molecular players and functional roles

### Canonical TGF-β signaling: ligands, receptors, and Smad effectors

#### TGF-β ligands (TGF-β1/2/3)

Structurally, all three isoforms (TGF-β1, TGF-β2, and TGF-β3) are synthesized as precursor proteins (pro-TGF-β), each containing an N-terminal latency-associated peptide and a C-terminal mature domain that forms a 25-kD homodimer. These precursors require extracellular activation via proteolytic cleavage, mechanical forces, and thrombospondin-1 binding for their biological activity. Bone tissue serves as a major reservoir of TGF-βs and harbors target cells essential for TGF-β activity.

Each TGF-β isoform exhibits a distinct expression pattern and plays a central role in bone ossification and homeostasis. TGF-β1 is abundantly expressed in osteoblasts, osteocytes, and chondrocytes; TGF-β2 is highly expressed in growth plate chondrocytes and the periosteum; and TGF-β3 is enriched in mesenchymal condensations and fracture callus. Among them, TGF-β1 is the most prevalent isoform in the bone matrix and is a dominant regulator of bone tissue.

Genetic studies underscore the non-redundant function of TGF-β isoforms. *TGF-β1*-knockout mice exhibit reduced bone growth and mineralization.^28^ *TGF-β2*-null mice experience perinatal mortality and widespread developmental defects,^29^ whereas double knockout of *TGF-β2* and *TGF-β3* causes defects in midline fusion and rib development.^30^ Conversely, osteoblast-specific overexpression of *TGF-β2* leads to progressive bone loss, characterized by increased osteoblastic matrix deposition and osteoclastic bone resorption,^31^ revealing its dual role in bone remodeling.

In vitro studies have further elucidated the multifaceted functions of TGF-βs. TGF-β3 robustly induces chondrogenesis in MSC while maintaining alkaline phosphatase (ALP) activity in the osteogenic layer during coculture with chondrocytes.^32^ Recombinant human TGF-β1 (rhTGF-β1) administration reverses denervation-induced impairment of bone formation rates and endochondral growth.^33^ Remarkably, a single-day TGF-β1 treatment induces chondrogenic differentiation in bone marrow stem cells (BMSCs),^34^ upregulates *bone sialoprotein and osteopontin*, and enhances ALP activity.^35^ However, TGF-β1’s role in osteoblastic differentiation is context-dependent, switching between induction and inhibition based on the Akt phosphorylation status.^36^ Clinically, the circulating levels of TGF-β1 and TGF-β2 have been identified as predictive biomarkers for bone non-union,^37^ emphasizing their clinical relevance. Thus, isoform-specific targeting may offer novel therapeutic strategies for improving bone repair.

### TGF-β receptors (Tgfbr1/2/3)

#### Core signaling receptors

TGF-β receptors (Tgfbr) exhibit distinct structural and functional properties, each playing unique roles in skeletal development and homeostasis. The core signaling complex consists of a type-I receptor (also known as ALKs) and a type-II receptor (Tgfbr2), whereas Tgfbr3 acts as an accessory receptor. Tgfbr2 (TGFβRII) is a constitutively active serine/threonine kinase that binds all TGF-β isoforms (TGF-β1/2/3), albeit with the highest affinity for TGF-β1.^38^ This process is essential for skeletal development and patterning.

Type-I receptors (ALKs) are pivotal in determining signaling specificity within TGF-β superfamily pathways: Tgfbr1 (Alk5) serves as the primary receptor responsible for phosphorylating Smad2/3; Alk1(AVRL1), though largely endothelial-specific, also contributes to the non-canonical Smad1/5/8 pathway within the bone vasculature^39^; and Alk2 (ACVR1), unique among type-I receptors in its ability to bind both activin and BMP ligands,^40^ plays a critical role in the pathogenesis of fibrodysplasia ossificans progressiva (FOP).

The type-III receptor, Tgfbr3 (betaglycan), lacks an intracellular kinase domain and does not directly transduce signals.^41^ Instead, it functions as a coreceptor that enhances ligand presentation to Tgfbr1 and Tgfbr2. Tgfbr3 binds all three TGF-β isoforms, but is particularly essential for potentiating TGF-β2 signaling, as this ligand exhibits 200–500-fold lower affinity for Tgfbr2 compared with TGF-β1 and TGF-β3.^42–44^ A key functional domain within TGF-β3, known as endoglin, plays a notable role in modulating the balance between Alk1- and Alk5-mediated signaling pathways.^42^

#### Genetic and functional insights of receptors

**Fig. 1:**
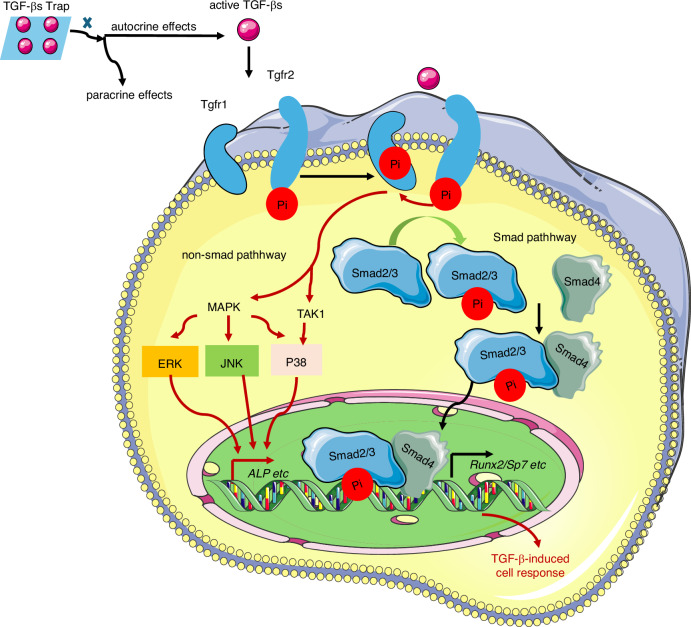
Molecular components of TGF-β signaling in bone. Canonical TGF-β signaling is mediated through the Smad-dependent pathway. Upon binding of TGF-β ligands to the type II receptor (Tgfbr2), Tgfbr2 recruits and phosphorylates the type I receptor (mainly Tgfbr1/ALK5). The activated type I receptor then phosphorylates receptor-regulated Smads (R-Smads, Smad2/3) at C-terminal SSXS motifs. Phosphorylated Smad2/3 dissociate from the receptor, form a heterotrimeric complex with Smad4, and translocate into the nucleus. Within the nucleus, this complex collaborates with lineage-specific transcription factors (e.g., Runx2, Sox9) and co-regulators (e.g., p300/CBP) to modulate the expression of target genes governing osteogenesis, bone matrix deposition, and skeletal homeostasis. In parallel, TGF-β signaling also activates non-canonical pathways, predominantly through TGF-β-activated kinase 1 (TAK1), which initiates downstream MAPK cascades (p38, JNK, ERK), which regulate osteogenic responses via both Smad-independent mechanisms and crosstalk with canonical Smad signaling. Phosphorylation (Pi) events at critical residues in receptors, Smads, and MAPKs serve as key regulatory switches, fine-tuning pathway activity and biological outcomes

Genetic studies have revealed the essential role of Tgfbr1 in skeletal biology. Mice with *Tgfbr1* deletion driven by *Dermo1-Cre* exhibit shortened and widened long bones, along with impaired formation of bone collars and trabeculae.^45^ *Tgfbr1* deficiency in cranial neural crest cells (CNCs) using *Wnt1-Cre* results in severe mandibular hypoplasia, loss of mandibular processes, and a three-fold reduction in osteoclast numbers,^46^ underscoring the necessity of Tgfbr1 in CNC survival, lineage specification, and skeletal patterning.^47^ Similarly, pharmacological inhibition of Tgfbr1 during the early mineralization and resorption stages leads to reduced jaw length in embryos.^46^ Notably, Tgfbr1 inhibition using SB505124 enhances bone formation in human BMSCs^48^ and improves trabecular bone architecture and mechanical properties in vivo.^49^ Conversely, mice expressing a constitutively active form of *Tgfbr1* (ca-Tgfbr1) driven by *Mx1-Cre* display decreased cortical thickness and cancellous bone volume, accompanied by increased osteoclast activity and reduced osteoblast numbers.^50^ Transcriptomic profiling of these models has revealed altered expression of bone turnover-related genes, identifying potential therapeutic targets for Tgfbr1-associated bone loss.^51^ (Fig. 1). Collectively, these findings indicate that Tgfbr1 exerts cell-type-specific and pleiotropic effects on skeletal biology.

The skeletal functions of Tgfbr2 have been elucidated using cell-specific deletion models. Mice with *Tgfbr2* deletion in MSCs using *Prx1-Cre* have exhibited defective long bones, joints,^52^ skull vault,^53^ and impaired endochondral growth.^54^ Ablation of *Tgfbr2* in the mesoderm-derived lineage via *Myf5-Cre* causes defects in the supraoccipital bone,^55^ whereas its deletion in cranial neural crest cells with *Wnt1-Cre* disrupts osteoprogenitor differentiation,^56^ leading to malformed craniofacial bones,^57^ including the premaxilla, maxilla, palatine bone, frontal bone, and mandibular bones, with complete penetrance in the cranial bone.^58^ Tgfbr2 is further required for sclerotome boundary establishment and axial skeleton patterning,^59^ as well as normal cranial and vertebral formation when deleted in chondrocytes using *Col2a1-Cre*.^60^ Mice expressing dominant-negative *Tgfbr2* (*dnTgfbr2*) exhibit cartilage hypoplasia,^61^ confirming its critical role in chondrogenesis.

Postnatally, *Tgfbr2* deficiency in *Gli1*^+^ skeletal stem cells (SSCs) reduces alveolar bone mass, mineral density (BMD),^62^ and root length^63^ and impairs endochondral ossification.^64^ *Tgfbr2* deletion in mature osteoblasts via *Osteocalcin-Cre* decreases cementum formation and mineralization.^65^ Conversely, the ablation of *Tgfbr2* in osteocytes using *DMP1-Cre* increases trabecular bone mass by 35% without altering the cortical bone,^66^ whereas deletion of *Nestin*^+^ stem cells mitigates joint degeneration.^67^ These studies demonstrate that Tgfbr2 is critically required for normal early skeletal development across diverse lineages, whereas its role in adult bone remodeling is highly context specific, influencing both anabolic and catabolic processes.

#### TGF-β receptor-dependent R-Smads (Smad2/3) signal transduction

TGF-β signaling primarily transduces its effects through the canonical Smad pathway. Upon ligand binding, Tgfbr2 transphosphorylates the type-I receptor ALK5, which subsequently phosphorylates Smad2 and Smad3 at their C-terminal SSXS motifs. Phosphorylated Smad2/3 plays an extensive and critical role in skeletal regulation.^68^

Genetic studies have highlighted the distinct and compensatory functions of Smad2 and Smad3. Smad2 is essential during early embryogenesis; homozygous *Smad2* mutant embryos die before E8.5, failing to gastrulate or form a mesoderm.^69^ Post-gastrulation deficiency results in impaired embryo turning and anterior morphogenesis,^70^ with the epiblast unable to generate the three germ layers.^71^ In contrast, *Smad3* mutant mice exhibit relatively normal skeletal development at younger ages but develop progressive defects with aging,^72^ suggesting a primary role in maintaining skeletal integrity, rather than formation. Conditional *Smad2-*knockout mice are viable and fertile with minimal skeletal alterations,^68^ whereas double *Smad2/Smad3*-knockout mice (*Smad2*^*cKO*^*/Smad3*^*−/−*^) show subtle neonatal defects, but severe postnatal dwarfism,^68^ underscoring functional redundancy in postnatal skeletal growth. In osteocytes, the TGF-β1-Smad2/3 axis is positively correlated with bone turnover parameters in subchondral bone.^73^ In embryos, Smad2/3-mediated TGF-β signaling modulates neural crest axial identity and directly controls the gene circuits that support skeletal differentiation.^74^ Notably, *Smad3*-knockout mice exhibit increased bone mass and improved matrix properties^75^ (Fig. 1, Table 1), suggesting that Smad3 inhibition may represent a viable strategy to enhance bone strength.

**Table 1:** Genetic alterations on TGF-β signaling components and their phenotypes

Gene	Cre	Defects	Reference
*TGF-β1*	knockout	reduced bone growth and mineralization	^28^
*TGF-β2*	knockout	perinatal mortality and wide developmental defects	^29^
*TGF-β2/β3*	knockout	a shortage of the distal parts of the rib	^30^
*TGF-β2*	*Osteocalcin-Cre*	progressive bone loss	^31^
*Tgfbr1 (ALK5)*	*Dermo1-Cre*	the short bones and ectopic cartilaginous protrusions	^45^
*caTgfbr1(ALK5)*	*Mx1-Cre*	decreased cortical thickness and cancellous bone volume; increased osteoclast number	^50^
*Tgfbr1 (ALK5)*	*Wnt1-Cre*	significantly shorter mandibles with no condylar, coronoid	^46^
*Tgfbr2*	*Prx1-Cre*	short limbs and fusion of the joints in the phalanges	^52^
	*Wnt1-Cre*	osteogenic cell proliferation and differentiation	^56^
	*Wnt1-Cre*	malformed craniofacial bones (premaxilla, maxilla, palatine bone, frontal bone, and mandible)	^57^
	*Wnt1-Cre*	defective skull with complete phenotype penetrance	^58^
	*Wnt1-Cre*	severe defects in mandibular development	^97^
	*Col2a1-Cre*	obvious defects in long bone formation	^61^
	*Col2a-Cre*	defects in the base of the skull and in the vertebrae	^60^
	*Myf5-Cre*	decreased chondrocyte proliferation and premature differentiation of cartilage to bone	^55^
	*Osteocalcin-Cre*	reduced matrix secretion and mineral apposition rates	^65^
	*Nestin-Cre*	cartilage and joint degeneration	^67^
	*DMP1-Cre*	increased trabecular bone mass but not cortical bone	^66^
	*Gli1-Cre (ERT2)*	significant reduction in alveolar bone mass and bone mineral density	^62^
	*Gli1-Cre (ERT2)*	a delayed and impaired endochondral bone formation	^64^
*Smad2/3*	*Col2a-Cre*	subtle skeletal alterations at birth, but progressive postnatal dwarfism	^68^
*Smad4*	*Tbx18-Cre*	a prominent shortened limb	^79^
	*Osx-Cre*	no alteration in osteoblasts number	^80^
	*Col2a1-Cre*	dwarfism	^82^
	*TTR-Cre*	early death with no head-fold and anterior embryonic structures	^78^
*caSmad7*	*Prx1-Cre*	poor cartilage formation	^98^

Following phosphorylation, Smad2/3 forms a trimeric complex with Smad4, which translocates to the nucleus via intrinsic nuclear localization signals.^76^ The Mad homology 1 (MH1) domain of Smad3 and Smad4 facilitates binding to Smad-binding elements (SBEs) in DNA^77^ and recruits lineage-determining transcriptional factors, such as Runx2, for osteoblast differentiation, Sox9 for chondrogenesis, and PPARγ for adipogenesis inhibition, establishing Smad4 as the central mediator of canonical TGF-β responses.

Genetic evidence highlights the non-redundant role of Smad4. Global *Smad4* deficiency in mice results in embryonic lethality between E7.5 and E9.5 due to failed anterior development and abrogated TGF-β responses.^78^ Limb mesenchymal deletion of *Smad4* via *Tbx18-Cre* results in limb shortening via the dysregulation of *Runx2* promoter activity.^79^ Osteoblast-specific *Smad4* deletion disrupts bone remodeling without affecting osteoblast numbers.^80^ Neural crest ablation of *Smad4* delays craniofacial morphogenesis,^81^ and chondrocyte-specific knockout impairs longitudinal bone growth^82^ (Table 1). These findings underscore the essential role of Smad4 as a positive regulator of TGF-β signaling in skeletal development and homeostasis.

#### Non-canonical TGF-β signaling pathway in bone

Beyond the canonical Smad-dependent pathway, TGF-β signaling exerts critical functions in the skeletal system through non-canonical signaling cascades, including p38 MAPK,^83^ JNK,^84^ ERK,^85^ and TGF-β-activated kinase 1 (TAK1).^86–88^ These pathways often intersect with Smad signaling at major transcriptional regulators, such as Runx2, to orchestrate mesenchymal stromal cell differentiation and skeletal morphogenesis.^89^ For instance, TGF-β1 induces maturation and mineralization in human osteoblast-like MG63 cells through JNK activation,^90^ whereas TGF-β2 promotes osteoblast differentiation through ERK-MAPK signaling, facilitating rapid calvarial bone expansion.^91^ Both ERK and p38 MAPK pathways mediate context-dependent responses to TGF-β and BMP2 in osteoblasts,^92^ highlighting their specialized roles in bone maintenance. TGF-β also stabilizes and phosphorylates Sox9 via p38 MAPK signaling,^93^ a critical mechanism in joint development. Notably, functional redundancy between p38 and Smad4 has been observed in embryonic organogenesis,^94^ further emphasizing the interplay between canonical and non-canonical TGF-β signaling (Fig. 1).

The TAK1-centered signaling module, comprising TRAF6, TAB1, and TAK1, plays an essential role in bone biology, serving as an upstream activator of p38 MAPK to stimulate type-I collagen synthesis^95^ and amplifies Smad signaling^87^ through direct interaction with Smad2/3.^88^ *TAK1* deficiency in CNCs results in craniofacial abnormalities,^86^ including a rounded skull morphology and a hypoplastic maxilla and mandible. Disrupted TGF-β signaling impairs the assembly of the TRAF6–TAB1–TAK1 complex,^88^ further linking non-canonical and canonical pathways. Moreover, the splicing factor Rbfox2 modulates the TGF-β-TAK1 axis, and its conditional deletion in CNCs results in deformed craniofacial bones.^96^ Collectively, these findings illustrate how non-canonical pathways confer signaling plasticity, enable rapid cellular responses, and provide compensatory routes under Smad-deficient conditions. Future investigations are essential for determining whether the patterns of MAPK-Smad crosstalk differ between the membranous and endochondral bones.

### TGF-β signaling crosstalk in osteoblast biology and bone homeostasis

#### Interplay between TGF-β and Wnt/β-catenin signaling in bone

The Wnt/β-catenin and TGF-β signaling pathways engage in dynamic, context-dependent crosstalk that plays a critical role in regulating osteoblast differentiation, skeletal development, and bone homeostasis.^5,20,97^ TGF-β has been shown to enhance Wnt/β-catenin signaling in MSC, thereby promoting osteoblast differentiation.^98^ For instance, siRNA-mediated knockdown of *Wnt/β-catenin* counteracts the inhibitory effect of TGF-β1 on bone sialoprotein expression, revealing a context where the two pathways act antagonistically.^99^ During endochondral ossification, members of the TGF-β family modulate Wnt/β-catenin signaling to regulate chondrocyte hypertrophy and vascular invasion.^100^ *Axin2*-mutant chondrocytes (constitutively active β-catenin) show amplified TGF-β-induced β-catenin stabilization,^101^ suggesting a positive feedback mechanism between the two pathways.

Transcriptional integration further underscores their interactions: TGF-β treatment induces *Wnt1* expression in osteoclasts,^102^ linking bone resorption and formation. Complexes formed between Smad3 and β-catenin are known to regulate osteogenic gene expression,^98^ although the stoichiometry and lineage-specific outcomes of this interaction remain unresolved. In *Gli1*^+^ mesenchymal cells, the deletion of *Tgfbr2* results in markedly reduced β-catenin levels and a sharp increase in *SOST* expression,^62^ a potent inhibitor of Wnt/β-catenin signaling, highlighting the essential role of TGF-β signaling in sustaining Wnt/β-catenin activity. Conversely, in a model of lumbar intervertebral disc degeneration, the activation of Wnt/β-catenin signaling using LiCl suppresses Smad-dependent transcription,^103^ revealing context-dependent antagonism. Additionally, TGF-β signaling combined with low Wnt/β-catenin signaling expands the potential of cranial neural crest cells to become skeletal elements.^74,104^ A key unresolved question is whether the combination with TGF-β mimetics and Wnt potentiators synergistically enhances bone regeneration beyond single-pathway modulation. The intricate interplay between TGF-β and Wnt/β-catenin signaling, encompassing both cooperative and competitive mechanisms, offers promising avenues for targeted skeletal therapies.

#### Interplay between TGF-β and PTH/PTHrP signaling in bone

PTH and PTH-related peptide (PTHrP) are pivotal regulators of extracellular phosphate and calcium homeostasis, as well as bone remodeling.^105–107^ Both ligands signal through a common G-protein-coupled receptor (PTH1R).^108,109^ The TGF-β and PTH/PTHrP pathways form an intricate regulatory network that coordinates bone remodeling, mineral homeostasis, and MSC fate determination. A key mechanism of interaction involves TGF-β-mediated phosphorylation of PTH1R, which triggers their co-internalization and leads to mutual attenuation of both signaling pathways.^110^ This feedback restraint helps to prevent excessive bone resorption and maintains the balance of remodeling. Osteoblasts lacking *Tgfbr2* exhibit hyperactive PTH1R signaling,^110,111^ leading to increased trabecular bone mass. This phenotype is rescued by either PTH (7-34) (antagonist) or the genetic deletion of *PTH1R*,^111^ confirming the functional interdependence of these pathways.

PTH signaling stimulates cyclic adenosine monophosphate (cAMP) production and phosphorylates the transcription factor cAMP response element-binding protein (CREB). The PTH-CREB pathway upregulates *BMP2* expression to stimulate osteoblast differentiation,^112^ linking catabolic (PTH) and anabolic (BMP) signaling. Intermittent PTH treatment also induces MCP1 secretion (monocyte chemoattractant protein-1),^113^ which amplifies TGF-β signaling and enhances bone remodeling. Furthermore, osteoclast-mediated bone matrix degradation releases latent TGF-β, which recruits MSCs to resorption sites,^114^ a process potentiated by PTH to ensure coupled bone formation.^114^ A central unsolved question is how TGF-β and PTH signals are integrated in a cell-type-specific manner (osteoblasts vs. MSC). Whether biased PTH1R ligands could be developed to avoid Tgfbr2-mediated co-internalization or a new intercellular linker protein to modulate its activity, thereby promoting bone formation without stimulating excessive resorption, remains an intriguing possibility. The TGF-β–PTH/PTHrP axis represents a master regulatory module in skeletal homeostasis and a promising target for novel anabolic therapies.

#### Interplay between TGF-β and FGF signaling in bone

FGFs and their receptors (FGFRs), comprising more than 20 ligands and 4 receptors, play essential roles in skeletal development.^115–117^ Both TGF-β and FGF signaling pathways act as potent inducers of osteoblast differentiation in vitro.^118^ Specifically, FGF2 synergizes with TGF-β1 to promote osteoblast differentiation in human umbilical cord perivascular cells.^119^ This synergy also enhances the migration and recruitment of BMSCs,^120^ processes critical for bone repair. In vivo, the co-delivery of FGF2, TGF-β1, and adipose-derived MSCs significantly improves healing in critical-sized femoral defects.^121^ Furthermore, FGFR3 in chondrocytes mediates the paracrine regulation of bone remodeling,^122^ highlighting its role in chondrocyte–osteoblast communication.

The interplay between TGF-β and FGF is highly context-dependent. For example, FGF2 enhances osteogenic differentiation while simultaneously repressing BMP6-induced chondrogenesis,^123^ underscoring its dose- and stage-specific effects. During posterior frontal suture fusion, TGF-β1 and FGF2 are co-expressed in the suture mesenchyme,^124^ and TGF-β-mediated FGF signaling is critically required for normal frontal bone development^125^ (Table 2). Additionally, FGF/FGFR3 signaling is essential for TGF-β’s effect on embryonic bone formation,^126^ and mutations in FGFR3 disrupt the TGF-β/FGF balance and impair bone growth. Whether FGFR3 inhibition can normalize bone growth in achondroplasia without adversely affecting TGF-β signaling is unresolved. Elucidating the dynamics of this crosstalk pathway may open new therapeutic avenues for skeletal dysplasia.

**Table 2:** TGF-β signaling interacts with other signals in osteoblasts

Gene	Crosstalk signaling	Results	Refererence
*Tgfbr2*↓	↑PTH type I receptor	increased bone mass	^112^
*Tgfbr2*↓	↓β-catenin ↑SOST	defective periodontal ligament	^62^
*Tgfbr1*↑	↓Hedgehog signaling	decreased cortical size	^50^
*Smad4 mutant*	Ihh/PTHrP↓	×→TGF-β1 response	^82^
*β-catenin siRNA*→	×TGF-β1 inhibitory effects	↑bone sialoprotein expression	^101^
*TGF-β*→	↑Axin2 mutant→Wnt signaling	endochondral ossification	^103^
*TGF-β*↑	↑Wnt1 expression	normal osteoclasts activity	^104^
*PTH*→	↑MCP1 release→↑TGF-β	supports bone remodeling	^114^
*PTH-CREB →*	↑BMP2 expression	osteoblastogenesis	^113^
*FGF2 ↑*	Tgfbr2 mutant→normal	regulating the frontal bone	^126^
*FGF/FGFR3*→	TGF-β	mediating bone formation	^126^
*TGF-β1*→	↑β-catenin	osteoblastogenesis ↑	^101^
*Wnt*→	↑TGF-β type I receptor	Runx2 ↑ osteoblast maturation ↑	^99^
*Smad4 mutant*	↓Ihh/PTHrP	×→TGF-β1 response	^82^
*TGF-β1*→	↑BMP2	→ectopic bone formation	^139^
*TGF-β*→	↑FGF signaling	normal bone development	^126^
*TGF-β1& FGF2*→	↑Bmpr1b expression	↑osteogenic differentiation	^137^
*TGF-β1*→	↑BMP2	→ectopic bone formation	^139^
*BMP6*→	↑TGF-β receptors	↑osteogenic differentiation	^138^
*TGF-β*→	↓canonical BMP signaling	↑Smad4 availability	^141^

↓decrease; ↑ increase; → stimulate; × block

#### Interplay between TGF and IGF/IGFR signaling in bone

The IGF system is a central regulator of skeletal growth and development.^127–130^ IGFs, TGF-βs, and BMPs are co-secreted by osteoblasts and other bone cells, where they act in concert to control osteoblast proliferation and differentiation.^131^ TGF-β1 and IGF1 serve as critical mediators of chondrogenic differentiation in MSCs.^132^ The co-overexpression of TGF-β1 and IGF1 via recombinant adeno-associated virus in MSCs significantly enhances the repair of osteochondral defects, improves proteoglycan/type-II collagen deposition, and promotes endochondral ossification.^133^ Additionally, collagen tripeptide 20 (CTP20) amplifies both the IGF1 and BMP signaling pathways, thereby stimulating longitudinal bone growth.^134^ Notably, the interaction between TGF-β1 and IGF1 is dose-dependent: low doses of TGF-β1 and IGF1 act synergistically to promote osteogenesis, whereas high doses can exhibit a suppressive interplay,^135^ suggesting the importance of precise dosing in therapeutic optimization.

#### Interplay between TGF-β and BMP signaling in bone

The TGF-β and BMP pathways exhibit complex, context-dependent interactions that are essential for skeletal development and regeneration. In vitro, TGF-β1, FGF2, and PDGF-AB upregulate *Bmpr1b* expression, thereby amplifying BMP2-induced osteogenic differentiation in MSCs.^136^ BMP6-driven osteogenesis similarly depends on functional TGF-β receptors,^137^ suggesting shared or interdependent receptor usage. In vivo, co-delivery with BMP2 and TGF-β1 generates a fivefold increase in bone volume over BMP2 alone.^138^ Furthermore, multiple type-II receptors, including BMPRII, ActRII, and ActRIIB, have been implicated in mediating the BMP9-induced osteogenic differentiation of C3H10T1/2 stem cells.^139^

Spatial antagonism between TGF-β and BMP signaling has also been observed. Specific deletion of *Tgfbr2* in cranial neural crest cells enhances canonical BMP activity (phospho-Smad1/5/8) in the posterior palate, but not in the anterior palate, owing to reduced competition between p-Smad2/3 and p-Smad1/5/8 for Samd4.^140^ The pharmacological inhibition of Tgfbr1 (e.g., with SB525334) similarly increases p-Smad1/5/8-Smad4 complex formation in the posterior palatal mesenchyme^140^ (Table 2). These studies emphasize the critical role of TGF-β-mediated interactions with BMP signaling in the regulation of osteoblast differentiation and bone formation.

## BMP signaling components in the skeletal system

### Canonical BMP signaling (ligands, receptors, Smads) in bone

#### BMP ligands

BMPs are pivotal regulators of cell proliferation, differentiation, and apoptosis, with functional outcomes highly dependent on the cellular context and ligand oligomerization states (homo-/heterodimers).^141^ Transcriptomic analysis has indicated that BMP genes are significantly enriched in biological processes, such as pathway-restricted Smad phosphorylation (92%) and cartilage development (77%).^142^ Although BMP1, BMP4, BMP5, and BMP6 are broadly expressed across tissues, BMP3, BMP10, and BMP15 display tissue-specific expression patterns.^142^ Among these, BMP2, BMP4, BMP7, and growth differentiation factor 5 (GDF5) play critical roles in embryonic skeletogenesis,^22,143^ whereas BMP6, BMP7, and GDF6 are essential for late-stage skeletal maturation.^22,143^

BMP2 is a potent inducer of osteogenesis, significantly upregulating osteocalcin^144^ and inducing bone formation, even under transient expression.^145,146^ It enhances *FoxO1* expression in BMSCs and promotes their proliferation, migration, and osteogenic differentiation.^147^ BMP2 signaling is temporally regulated in response to mechanical loading, thereby linking mechanical loading to cytoskeletal remodeling.^148^ During limb development, BMP2 is not directly involved in early patterning, but is essential for initiating mesenchymal condensation prior to ossification and for mediating apoptosis.^149^ Conditional deficiency of *BMP2* in CNCs via *Wnt1-Cre*^149,150^ leads to craniofacial anomalies due to the disrupted coordination between osteoprogenitor proliferation and differentiation.^150^ A conserved ultra-conserved sequence (UCS) within the 3′UTR of BMP2 critically regulates its mRNA stability and signaling output.^151^ *UCS* deletion results in elevated *BMP2* expression, hyperactive BMP signaling, and embryonic abnormalities.^151^ Therapeutically, targeting BMP2 effectively stimulates osteogenesis, and the BMP2/4 consensus peptide (BCP) has emerged as a promising candidate for full-length BMP2 to induce osteoblast differentiation.^152^

BMP7 stimulates osteoblastic differentiation and calcium mineralization.^153,154^ Although locally produced BMP7 is dispensable for postnatal limb growth,^155^ it is essential for appendicular skeletal development.^156^ Similarly, the absence of *BMP4* does not impair normal limb skeletogenesis in mice,^157^ with BMP2, rather than BMP4, identified as the primary regulator of chondrocyte proliferation and maturation.^158^ Nonetheless, BMP4 is essential for craniofacial cartilage patterning,^159^ hindlimb growth,^160^ and cleft lip development.^161^ Double knockout of *BMP2* and *BMP4* severely impairs osteogenesis.^162^ Secreted from cranial neural crest cells or mesoderm-derived preosteoblasts and dura, BMP2/4 acts in a paracrine manner to regulate cerebral vein patterning^163^; mutations in these ligands disrupt both cerebral vein formation and skull development.^163^

In contrast, *BMP3*-mutant mice exhibit a two-fold increase in trabecular bone volume.^164^ *BMP3* overexpression reduces BMP signaling and expands skeletal elements by enhancing cell proliferation.^165^ BMP3 attenuates BMP signaling via Acvr2b in skeletal progenitor cells, thereby restricting their differentiation into mature osteoblasts.^166^ Transgenic mice overexpressing *BMP3* develop spontaneous rib fractures and exhibit altered ActRIIB signaling in the chondrocytes and periosteum.^167^ BMP3b (GDF10) inhibits osteoblast differentiation and acts as a mutual antagonist of BMP2, partly by competing for Smad4 availability^168^ or acting as a BMP receptor antagonist.^169^ Similarly, GDF11 inhibits the osteoblastic differentiation of BMSCs in vitro.^170^ *GDF6*, which is expressed in the primordia of the frontal bones, is critical for cranial development, as its deficiency results in fused coronal sutures and defective frontal and parietal bones.^171^ Collectively, these findings underscore the diverse, context-specific, and often antagonistic roles of BMP ligands in skeletal development and homeostasis.

#### BMP receptors

TGF-βs, BMPs, and activins signal through heterotetrameric complexes composed of type-I and -II serine/threonine kinase receptors.^172,173^ This architecture allows for a limited repertoire of ligands to elicit diverse biological responses through combinatorial assembly and context-dependent interpretations. BMP receptors typically comprise two type-II receptors (e.g., BMPRII, ActRIIA, and ActRIIB) and two type-I receptors (Alk1-3 and Alk6). Type-II receptors are constitutively active serine/threonine kinases that phosphorylate and activate type-I receptors, which in turn phosphorylate receptor-regulated Smads (R-Smads). This modular system enables the 33 known ligands to generate cell- and tissue-specific outcomes through selective receptor pairing,^174,175^ differential binding affinities influenced by ligand structural domains,^176^ and varying downstream signal integration.

**Fig. 2:**
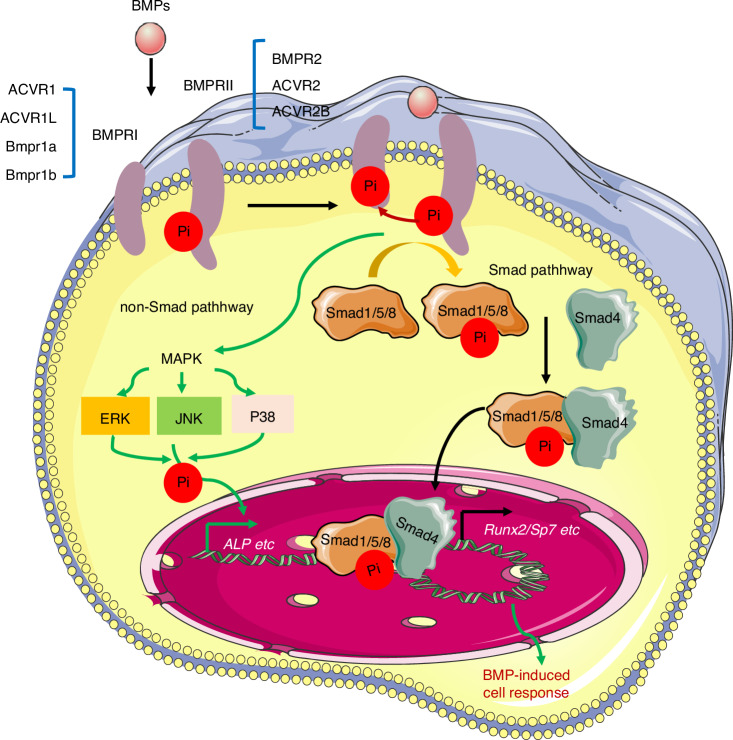
The components of BMP signaling pathways in bone. BMP signaling is initiated when ligands (e.g., BMP2/4/6/7/9) bind to constitutively active type II receptors (BMPR2, ACVR2A, or ACVR2B). This promotes recruitment and transphosphorylation of type I receptors (Alk2, Alk3, or Alk6), which in turn phosphorylate receptor-regulated Smads (R-Smads: Smad1/5/8). Phosphorylated R-Smads form a complex with the common mediator Smad4, and this heteromeric complex translocates into the nucleus. There, it associates with lineage-determining transcription factors (e.g., Runx2, Osterix) and co-regulators (p300/CBP) to modulate expression of osteogenic target genes. Concurrently, non-canonical pathways are activated, most notably the MAPK cascades (ERK, JNK, p38), which contribute to the regulation of osteoblast proliferation, differentiation, and mechanical adaptation. Phosphorylation (Pi) events at key residues in receptors, Smads, and MAPKs act as critical regulatory switches that finely modulate signaling intensity and downstream biological outcomes

Ligand–receptor affinity is a key determinant of signaling specificity.^174,175^ BMPRII selectively binds to BMPs, whereas activin type-II receptors (ActRIIA/ACVR2A and ActRIIB/ACVR2B) interact with both BMPs and activins. Type-I receptors include ALK1 (ACVRL1), Alk2 (also known as ActRI or ACVR1), Alk3 (Bmpr1a), and Alk6 (Bmpr1b).^177^ BMP2 and BMP4 exhibit high binding affinities for Alk3 and Alk6, whereas BMP6 and BMP7 preferentially bind to Alk2. BMP9 and BMP10 are high-affinity ligands for Alk1, but can also signal through ALK2^177^ (Table 3). GDF5 acts primarily through Bmpr1b (Alk6) in vivo, but exhibits functional overlap with other BMP receptors during skeletal development, highlighting both redundancy and specificity within the pathway^156^ (Fig. 2, Table 3). Recent structural studies have elucidated the key features of BMP–receptor interactions,^178^ providing new insights into the initial events of BMP–receptor activation. However, key questions remain unsolved, such as how ligand–receptor binding kinetics and oligomerization states influence signaling duration and strength, and can these parameters be therapeutically modulated to achieve precise control of bone formation and repair? Addressing these challenges is essential for developing novel strategies to manipulate BMP signaling in skeletal disorders.

**Table 3:** Characterized BMP receptor-ligand interactions

BMP receptor	Subtypes	High-affinity ligands
Type II	*BMPRII*	*BMP2/4/7*
	*ActRIIA/ACVR2A*	*BMP7, Activins*
	*ActRIIB/ACVR2B*	*BMP7, Activins*
Type I	*Alk1 (ACVRL1)*	*BMP9/10*
	*Alk2 (ACVR1)*	*BMP6/7, Activins*
	*Alk3 (Bmpr1a)*	*BMP2/4*
	*Alk6 (Bmpr1b)*	*GDF5, BMP4*

#### Type-II BMP (BMPRII) receptor

BMPRII plays a critical role in modulating the responsiveness of target genes to BMP2/4/7.^179^ Both BMPRII and ActRIIB exhibit functional redundancy, enabling the compensatory activation of BMP2 signaling and the coordinated regulation of osteoblast differentiation.^180^ Interestingly, BMPRII deficiency in the early limb mesoderm via *Prx1-Cre* does not impair skeletal development, suggesting that BMPRII is dispensable for limb development, or that its absence may be functionally compensated for by other type-II receptors.^181^

ActRIIA and ActRIIB, which bind ligands including BMP7 and Activins, have emerged as promising therapeutic targets for modulating bone homeostasis. The soluble extracellular domains of these receptors have been engineered to act as ligand traps, effectively blocking downstream signaling. Mice treated with modified ActRIIA have shown enhanced bone formation and increased bone mass,^182^ whereas those treated with modified ActRIIB have shown reduced osteoclast activity, enhanced bone formation,^183^ and mitigated bone loss in ovariectomized mice.^184^ Despite these advances, the precise genetic functions of ActRIIA and ActRIIB in the skeletal system remain unclear, warranting further investigation to elucidate their cell-type-specific functions and regulatory mechanisms in both physiological and pathological contexts.

### Type-I BMP receptors (ALKs)

#### Alk2 (ACVR1) receptor

Activin A receptor type 1 (ACVR1, also known as Acvr1 or Alk2), a BMP type-I receptor, shows selective ligand binding to BMP6/7 and Activin A, and plays a critical role in skeletal development. Cryo-EM structural analysis of the Alk2–BMP6 complex revealed a unique mechanism that enables Alk2 to engage both BMP and activin ligands.^40^ Alk2 functions as a hybrid receptor, combining the structural features of BMP type-I receptors (such as Alk3) at the wrist interface with those of activin type-I receptors (such as Alk4) in the fingertip region.^40^

Heterozygous mutations in *ACVR1* are responsible for all cases of FOP,^185^ a rare genetic disorder characterized by congenital malformations of the big toes and progressive heterotopic ossification (HO) in soft connective tissues via endochondral ossification. The most prevalent mutant variant, Alk2 p.R206H, leads to the loss of autoinhibition of BMP signaling and confers constitutive activation in response to activins, a response not observed in wild-type receptors.^185^ Postnatal expression of the human mutant *Alk2 (Alk2-R206H)* in mice recapitulates an FOP-like phenotype, further confirming the central role of dysregulated Alk2 signaling in FOP pathogenesis.^186^ RK783 is a novel small-molecule inhibitor designed to selectively target the activity of BMP type-I receptors. Developed as a safe and efficient oral treatment, it has been found to robustly suppress ectopic bone formation in a mouse model of FOP,^187^ demonstrating high therapeutic potential for clinical translation.

Mice lacking *Alk2* in the cranial neural crest cells via *Wnt1-Cre* exhibit craniofacial defects,^188^ including cleft palate and mandibular hypoplasia. The *Acvr1(R206H)* mutation generated in *Prx1-Cre* knock-in mice disrupts joint specification, impairs digit separation, and alters joint morphogenesis,^189^ highlighting Alk2’s role in joint development. Enhanced Alk2 signaling accelerates chondrocyte differentiation during early chondrogenesis.^190^ Constitutive activation of *Alk2 (caAlk2)* induces the ectopic phosphorylation of Smad1/5/8^191^ and promotes chondrogenesis over osteogenesis in cranial neural crest cells^192^ while also suppressing glycolytic activity via p53-mediated lactate reduction,^193^ leading to severe midline facial defects. In chondrocytes, *caAlk2* activation via *Col2-Cre* causes neonatal lethality and severe craniofacial abnormalities, such as enlarged nasal cartilage.^194^ Furthermore, *caAlk2* expression induces ectopic cartilage and bone formation in distal joints.^195^ In osteoclasts, *Alk2* deficiency using *Ubi-CreTM* reduces demineralization,^196^ whereas caAlk2 activation enhances osteoclast resorptive activity^196^ and promotes osteoclastogenesis in an osteoclast-autonomous manner, leading to reduced bone volume in mice.^197^

The phenotypic outcomes of *caAlk2* activation are highly dependent on the genetic background and the specific Cre driver lines used. For example, in cranial neural crest cells, *caAlk2* activation using line L35 results in ectopic cartilage and facial hypoplasia, whereas line A11 causes cleft palate and mandibular shortening.^198^ In the limb buds, *caAlk2* activation using line A11 produces smaller skeletal structures, whereas line L35 leads to disorganized limb patterning with minimal mineralization.^198^ Notably, *caAlk2* activation using *NFATc1-Cre* in line A11 does not induce HO, whereas line L35 displays robust HO, emphasizing the context-dependent nature of Alk2 signaling.^198^ Tmem150b has been identified as a negative regulator of BMP signaling through physical interaction with Alk2.^199^ Collectively, these findings underscore the pivotal and context-specific roles of Alk2 in skeletal development and disease, revealing its importance as a therapeutic target in disorders involving dysregulated bone and cartilage formation.

#### Bmpr1a (Alk3) receptor

Bmpr1a (Alk3), a high-affinity receptor for BMP2 and BMP4, is one of the most extensively studied BMP type-I receptors and plays a critical role in diverse developmental processes. Bmpr1a (Alk3) expression is precisely regulated during embryogenesis; it is detectable in the anterior palatal shelves from E13.5 and in the posterior palatal shelves from E14.5,^200^ where it is indispensable for hindbrain neural tube closure.^200^ In developing limbs, Bmpr1a is essential for the formation of mesenchyme-derived bone elements that define bone shape and size.^201^

Genetic studies using conditional mouse models have revealed a broad phenotypic spectrum resulting from *Bmpr1a* manipulation. *Bmpr1a* deficiency in the limb mesenchyme results in severe limb defects and autopod agenesis,^202^ whereas its ablation in auricular chondrocytes leads to chondrocyte atrophy and microtia.^203^ Bmpr1a is also required for proper skull development.^204^ Osteocyte-specific deletion using *Dmp1-Cre* reduces overall osteoblast activity, but accelerates preosteoblast proliferation in cancellous bone.^205^ Although joint formation appears to be normal in *Gdf5-Cre-*driven knockout mice, the articular cartilage progressively deteriorates,^206^ leading to OA-like phenotypes. In the maxillary mesenchyme, *Bmpr1a* deletion via *Nestin-Cre* results in decreased cell proliferation and defective anterior–posterior patterning.^161^ During dental development, Bmpr1a regulates odontoblast differentiation through a BMP-dependent transcriptional program, and its deficiency via *Gli1-Cre* disrupts root development.^207^

Constitutive activation of *Bmpr1a* in the palatal mesenchyme induces a complete cleft palate owing to enhanced BMP-Smad4 signaling and premature osteogenic differentiation.^208^ Conversely, *Bmpr1a* knockout in mice using *Col1-Cre* increases bone volume, but markedly decreases bone formation rates in the tibias.^209^ Other reported phenotypes include shortened limbs, autopod agenesis,^202^ reduced body size, irregular calcification, and low bone mass.^210^ Loss of *Bmpr1a* also increases trabecular bone mass by decreasing osteoclastogenesis through the modulation of the RANKL-OPG axis.^211^ Interestingly, hemizygous constitutive activation of *Bmpr1a* (caBmpr1a, wt/+) in osteoblasts using *Osx-Cre* elevates BMP signaling without altering adult bone mass,^212^ suggesting that mature osteoblasts are resilient to changes in BMP pathway activity, which has important implications for the clinical use of BMPs, particularly in terms of dosage and side effects.

Cranial bones and sutures originate from CNC cells, where Bmpr1a plays a vital role. The deletion of *Bmpr1a* in CNCs using *P0-Cre* results in 100% abnormal phenotypes, including wide-open anterior fontanelles.^213^ Similarly, *Wnt1-Cre-*mediated ablation leads to malformation of the temporomandibular joints.^214^ The constitutive activation of *Bmpr1a* in CNCs induces craniosynostosis,^201^ partially through mTOR-Gli1 hyperactivation in suture stem cells,^215^ and triggers p53-mediated apoptosis in nasal structures.^216^ Collectively, Bmpr1a is a central regulator of skeletal development that influences morphogenetic processes in limb and craniofacial patterning, maintains tissue homeostasis in bone remodeling and joint integrity, and contributes to pathologies such as craniosynostosis, OA, and dental defects.

#### Bmpr1b (Alk6) receptor

Bmpr1b (Alk6) plays critical and stage-specific roles in skeletal development via dynamic expression patterns and selective ligand interactions. It exhibits strong spatiotemporal expression, with a prominent presence in the early cartilage primordium and perichondrium at E14, followed by a sharp decline upon the onset of chondrocyte (pre)hypertrophy at E16.^217^ Bmpr1b has high affinity for GDF5 and BMP4.

Endogenous Bmpr1b signaling is essential for early osteoblast differentiation in vitro^218^ and is required for the BMP2-mediated expression of osteogenic genes such as *ALP, Dlx5*, and *Runx2* in bone cells.^219^ The inhibition of Bmpr1b via siRNA reduces osteoclastogenesis by modulating the RANKL-OPG pathway,^220^ suggesting its anti-resorptive role. BMP7 signals through ALK-6/Bmpr1b to halt terminal chondrocyte differentiation,^217^ and double knockout of *Bmpr1b* and *BMP7* results in severe appendicular skeletal defects,^156^ highlighting the functional interplay between these two pathways (Fig. 2, Table 4). Additionally, the BMP2-Bmpr1b-ODAM-MAPK signaling cascade has been identified as a significant regulator of enamel mineralization.^221^

**Table 4:** Molecular functions of BMP signaling components in skeletalogenesis

Gene	Cre	Defects	Refererence
*BMP2*	*Wnt1-Cre*	smaller craniofacial bones	^151^
*BMP2/BMP4*	*Wnt1-Cre*	defective skull and dural cerebral veins	^164^
*BMP7*	*Prx1-Cre*	normal postnatal limb growth	^156^
*BMP7*	*knockout*	appendicular skeletal defects	^157^
*BMP2*	*Prx1-Cre*	spontaneous fractures	^147^
*BMP2/BMP4*	*Col2a1-Cre*	a severe chondrodysplasia phenotype	^159^
*BMP2/BMP4*	*Col2a1-Cre*	a severe chondrodysplasia phenotype	^159^
*BMP4*	*Prx1-Cre*	normal limb skeletogenesis	^158^
*BMP2/BMP4*	*Prx1-Cre*	severely impaired osteogenesis	^163^
*BMP4*	*Isl1-Cre*	hindlimb fusion and pelvic/urogenital organ dysgenesis	^161^
*BMP4*	*Nestin-cre*	isolated cleft lip	^162^
*BMP7*	*Wnt1-Cre*	alteration of oral cavity morphology	^238^
*BMP7/BMP4*	*Mef2c-Cre*	defective mesenchymal transition	^239^
*BMPRII*	*Prx1-Cre*	normal skeletons	^182^
*Alk2 (ACVR1)*	*Wnt1-Cre*	multiple craniofacial defects	^189^
*Alk2 (ACVR1)*	*Wnt1-Cre*	impaired neural-crest cells	^240^
*Alk2 (ACVR1)*	*Ubi-Cre*^*TM*^	reduced osteoclast numbers and demineralization	^197^
*Alk2 (R206H)*	*Prx1-Cre*	disrupted joint specification/endochondral ossification	^190^
*caAlk2*	*Nfatc1-Cre*	ectopic cartilage and bone at the distal joints	^196^
*caAlk2*	*Col2-Cre*	severe craniofacial abnormalities	^195^
*caAlk2*	*Ubi-Cre*^*TM*^	enhanced osteoclastogenesis	^197^
*caAlk2*	*P0-Cre*	shorter jaws in line A11; ectopic cartilages in line L35	^199^
*caAlk2*	*P0-Cre*	drastic midline facial defects	^194^
*caAlk2*	*Prx1-Cre*	smaller limb structures in line A11; disorganized limbs with little mineralization in line L35	^199^
*caAlk2*	*NFATc1-Cre*	no heterotopic ossification (HO) in line A11; developed HO around the ankle joint in line L35	^199^
*caAlk2*	*Ctsk-Cre LysM-Cre*	enhanced osteoclastogenesis and reduced bone volume	^198^
*Bmpr1a (Alk3)*	*Wnt1-Cre*	post-migratory development of CNC-derived cell types	^201^
*Bmpr1a (Alk3)*	*Pax3-Cre*	reduction in neural-crest cells	^201^
*Bmpr1a (Alk3)*	*Wnt1-Cre*	defective temporomandibular joint	^215^
*Bmpr1a (Alk3)*	*P0-Cre*	wide-open anterior fontanelles	^214^
*Bmpr1a (Alk3)*	*Prx1-Cre*	severe limb defects	^203^
*Bmpr1a (Alk3)*	*Prx1-Cre*	led to chondrocyte atrophy and microtia development	^204^
*Bmpr1a (Alk3)*	*Dmp1-Cre*	diminished periosteal bone growth juxtaposed with excessive cancellous bone formation	^206^
*Bmpr1a (Alk3)*	*Dermo1-Cre*	defective ventral body wall formation	^241^
*Bmpr1a (Alk3)*	*Col1a1-Cre*	increased bone volume	^210^
*Bmpr1a (Alk3)*	*Prx1-Cre*	shortened limbs and almost complete agenesis of autopod	^203^
*Bmpr1a (Alk3)*	*Col1-CreER*	increased bone mass	^242^
*Bmpr1a (Alk3)*	*Col1-CreER*	increased bone mass/decreased osteoclastogenesis via RANKL-OPG	^212^
*Bmpr1a (Alk3)*	*Gli1-CreER*	impaired root development	^208^
*Bmpr1a (Alk3)*	*Nestin-Cre*	diminished cell proliferation in the maxillary process mesenchyme	^162^
*Bmpr1a (Alk3)*	*Gdf5-Cre*	joints gradually wear away after birth	^207^
*Bmpr1a (Alk3)*	*Osr2-Cre (KI)*	premature osteogenic differentiation in palatal MSCs	^209^
*caBmpr1a*	*P0-Cre*	craniosynostosis	^202,215^
*caBmpr1a*	*Osx-Cre*	no overt skeletal changes in adult mice	^213^
*Bmpr1b/BMP7*	*knockout*	severe appendicular skeletal defects	^157^
*Noggin*	*Osr2-Cre (KI)*	repressed osteogenic condensation in palatal MSCs	^209^
*Smad1*	*Col1a1-Cre*	osteopenic phenotype	^229^
*Smad1*	*Col2a1-Cre*	calvarial bone development delay	^229^
*Smad1/5*	*Col2a1-Cre*	chondrodysplasia	^230^
*Smad4*	*Wnt1-Cre*	defective mid-gestation	^232^
*Smad4*	*Wnt1-Cre*	underdevelopment of the branchial arch	^81^
*Smad4*	*TTR-cre*	died without a head-fold and anterior structures	^78^
*Smad4*	*Mu-Cre*	misalignment of the cardiac outflow tract	^243^

Genetic evidence has underscored the importance of Bmpr1b in skeletal development. Double mutants of *ACVR1/Bmpr1a* and *ACVR1/Bmpr1b* exhibit perinatal lethal chondrodysplasia,^222^ with a phenotypic severity exceeding that of single mutants,^222^ indicating that ACVR1 coordinates with both Bmpr1a and Bmpr1b to regulate skeletal development. Therapeutically, the administration of recombinant Bmpr1b-Fc protein increases bone volume in wild-type mice,^223^ suggesting its potential utility in treating bone-related disorders. Collectively, Bmpr1b’s stage-specific expression and interactions with multiple ligands are potential therapeutic targets for skeletal disorders. Future studies should explore the precise mechanisms underlying Bmpr1b regulation, develop receptor-specific modulators, and design tissue-specific delivery strategies for clinical application.

#### Intracellular canonical Smads

BMP signaling plays a pivotal role in modulating pathway-restricted Smad phosphorylation, decisively regulating over 90% of the biological processes associated with skeletal formation.^142^ This canonical cascade governs osteogenesis, chondrogenesis, and craniofacial patterning through the spatiotemporally precise activation of receptor-activated Smads (R-Smads, Smad1/5/8) and their common mediator, Smad4.^224^ BMP ligands induce the C-terminal phosphorylation of Smad1/5/8^225^ via the type-I receptor (e.g., ALK2),^191^ a critical step in downstream signal transduction.^226,227^

Smad1 is essential for bone growth, and its deficiency disrupts calvarial bone formation,^228^ osteoblast-specific deletion causes osteopenia and blunts BMP responses.^228^ Triple-knockout of *Smads1/5/8* causes severe chondrodysplasia, underscoring the functional redundancy among these R-Smads.^229^ A rare missense mutation in *Smad9* (c.65T>C, p. Leu22Pro) causes elevated bone mass,^230^ establishing Smad9 as a novel high bone mass gene and potential osteoanabolic target. Smad1 also shows non-canonical functions, such as stabilizing p53 by inhibiting MDM2-mediated degradation,^216^ thereby linking BMP signaling to apoptosis in the nasal cartilage.

Phosphorylated Smad1/5/8 forms heterotrimeric complexes with Smad4 and translocates to the nucleus, where it regulates its transcription. Smad4, the central node for both TGF-β and BMP signaling, is indispensable for skeletal homeostasis. Deficiency of *Smad4* in osteoblasts reduces bone mineral density, volume, and formation rates owing to impaired osteoblast function.^76^ In cranial neural crest cells, *Smad4* ablation results in neonatal lethality, increased apoptosis,^231^ and branchial arch defects^81^ (Table 4).

The regulation of Smad4 ubiquitination by Ectodermin/Tif1gamma (Ecto) and its deubiquitylation by FAM represent novel mechanisms that modulate BMP signaling and skeletal development.^232,233^ Smad6, an inhibitory Smad, fine-tunes BMP signaling by binding to R-Smads and forming transcriptionally inactive complexes with Smad4,^234^ creating a negative feedback loop.^235^ A rare inherited frameshift mutation in *Smad6* (p.152 fs*27) is linked to non-syndromic craniosynostosis.^236^ Collectively, canonical BMP-Smad signaling is highly dynamic, rather than linear, with inhibitory Smads and ubiquitin modifiers acting as tunable rheostats to ensure the precise control of skeletal development and homeostasis. These findings highlight promising therapeutic targets for bone-related disorders.

#### Non-canonical BMP signaling in bone

Although canonical BMP signaling primarily functions through Smad1/5/8 phosphorylation, emerging evidence highlights the critical importance of Smad-independent (non-canonical) pathways in skeletal biology. These signaling cascades, predominantly involving the p38, ERK1/2, and JNK MAPK pathways,^237^ provide nuanced regulation of osteogenic processes.

TAK1 acts as a central node in BMP–MAPK crosstalk.^237^ *TAK1* deficiency in osteoblasts leads to striking skeletal abnormalities, including clavicular hypoplasia and delayed fontanelle fusion, which are phenotypes that closely resemble human cleidocranial dysplasia caused by RUNX2 haploinsufficiency.^83^ Mechanistically, TAK1 orchestrates osteoblast differentiation through the TAK1–MKK3/6–p38 MAPK axis, which phosphorylates Runx2 and enhances its transcriptional activity by facilitating its interaction with the coactivator CREB-binding protein (CBP)/p300.^83^

Notably, *TAK1* deletion produces a dual signaling effect by disrupting p38 MAPK activation and simultaneously amplifying Smad1/5/8 phosphorylation.^238^ This suggests the existence of cross-inhibition between the MAPK and Smad pathways under physiological conditions, potentially mediated by MAPK phosphatases that modulate Smad activity. These findings reveal a sophisticated balance between canonical and non-canonical BMP signaling during bone development.

Ordination between Smads and the MAPK pathway is crucial during limb development.^239^ The osteogenic program requires the precise integration of TGF-β/BMP2-activated Smads, MAPK-mediated phosphorylation events, Runx2 subnuclear targeting, and transcriptional activation^240^ (Fig. 2). This multi-layered regulation ensures spatiotemporal precision in bone formation, and the convergence of these signals on Runx2 provides a mechanistic basis for how SSCs decode complex extracellular cues to drive skeletal morphogenesis.

## BMP signaling crosstalk in osteoblast biology and bone homeostasis

### Transcriptional regulation of osteogenesis: role of Runx2, Dlx5, and Osx in bone

Runx2 is a master transcription factor that is essential for both endochondral and intramembranous ossification. *Runx2*-null mice exhibit a complete absence of bone formation.^241^ It is unequivocally required for embryonic ossification,^207^ postnatal skeletogenesis,^242–244^ and craniofacial patterning.^245,246^ Runx2 activity is tightly linked to BMP signaling; autocrine BMPs activate *Runx2* expression,^247^ BMP2-induced osteogenesis strictly depends on Runx2,^248^ and Smad1 directly binds to the *Runx2* promoter to regulate osteoblast-specific gene expression.^249^ Heterozygous mutations in *RUNX2* cause cleidocranial dysplasia, an autosomal-dominant skeletal disorder.^250^

**Fig. 3:**
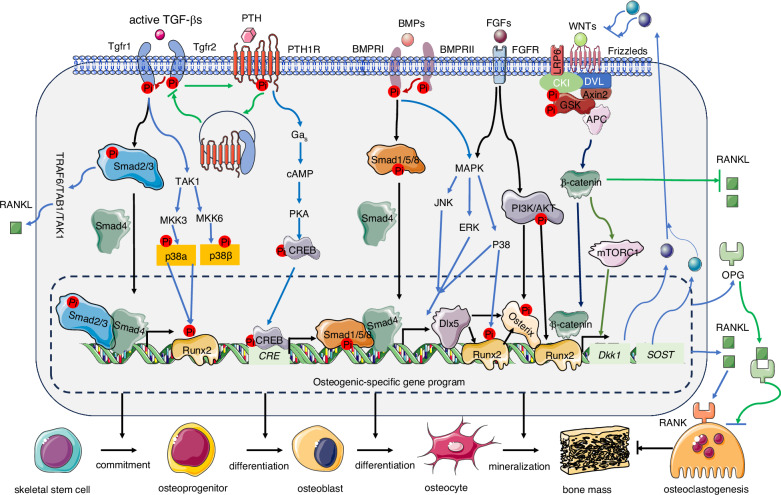
Signaling network integration in osteogenesis: TGF-β/BMP crosstalk with key bone regulatory pathways. TGF-β and BMP signaling pathways form a highly interconnected regulatory network with FGF, Wnt/β-catenin, PTH/PTH1R, and MAPK cascades to coordinately orchestrate osteoblast differentiation and bone formation. PTH signaling exhibits dual functionality through PTH1R: (1) canonical Gαs/cAMP/CREB activation promotes osteogenesis, while (2) ligand-induced internalization of PTH1R-TGFβRII complexes establishes a negative feedback loop that downregulates both PTH and TGF-β signaling. TGF-β-activated Smad2/3 physically interacts with the TRAF6-TAB1-TAK1 complex (induced by RANKL stimulation), creating a molecular bridge that links osteoblast and osteoclast activities. These Smad complexes transcriptionally regulate osteogenic master regulators (e.g., Dlx5, Runx2, and Osx). BMP signaling modulates the Wnt pathway through (i) Smad-dependent upregulation of *Sost (sclerostin)*, and (ii) both Smad-dependent and Smad-independent (e.g., p38 MAPK) induction of *Dkk1* expression. These secreted inhibitors suppress canonical Wnt signaling, thereby influencing bone mass regulation via the RANKL/OPG axis

Dlx5 acts as a BMP-responsive mediator and functions upstream of Runx2 in cranial sutures.^251^ Dlx5 cooperates with Mef2 to activate the *Runx2* enhancer,^252^ thereby driving osteoblast-specific gene expression. Osterix (Osx/Sp7) is another critical executor of osteoblast terminal differentiation, and its null mice lack bone entirely, despite normal *Runx2* expression.^10,253–256^ Although Osterix was initially identified as a Runx2-targeted gene, BMP2-induced *Osterix* expression has been found to be mediated by Dlx5, rather than by Runx2, as demonstrated by the abolition of this induction upon *Dlx5* antisense treatment.^257^ Post-translationally, the serine/threonine kinase Akt phosphorylates both Osterix and Dlx5,^258^ enhancing their stability and osteogenic activity^259^ (Fig. 3). An intriguing question is why certain BMPs preferentially signal via the Dlx5–Osx axis, whereas others operate via the Runx2 axis. These findings highlight the intricate regulatory network integrating BMP signaling, Runx2, Osterix, Dlx5, and Akt in orchestrating osteoblast differentiation and skeletal development.

### Interplay between BMP, Wnt/β-catenin, and mTOR signaling in osteoblast and bone

The functional synergy between BMP and Wnt/β-catenin signaling is critical for osteogenesis. β-catenin is essential for cellular responsiveness to BMP2,^260^ and Wnt3A and BMP9 synergistically enhance ALP activity in MSCs via Runx2–β-catenin interaction.^261^ Axin2 serves as a molecular bridge connecting Wnt/β-catenin and BMP signaling via the β-catenin–BMP2/4–Osterix axis.^262^ Feedback regulation further fine-tunes this crosstalk. *Bmpr1a* deficiency in osteoblasts leads to hyperactivated Wnt/β-catenin signaling and increased bone mass,^263^ resulting from the downregulation of the Wnt/β-catenin antagonists *sclerostin (SOST)* and *DKK1*.^205^ The ectopic expression of *Wnt7b* fully rescues periosteal bone growth in *Bmpr1a*-deficient mice.^264^ Both SOST and DKK1 act as downstream effectors of BMP signaling that inhibit canonical Wnt/β-catenin activity, thereby negatively regulating bone mass.^265^ Loss of either *DKK1* or *SOST* results in high bone mass phenotypes in both humans and mice,^266^ underscoring their critical roles in bone homeostasis (Fig. 3, Table 5).

**Table 5:** BMP signaling crosstalk in osteoblasts: integration with key regulatory pathways

Gene	Crosstalk signaling	Results	Refererence
*β-catenin*→	↑Runx2→BMP9	regulating osteogenesis	^268^
*Axin2*→	↑β-catenin-BMP2/4-Osterix	regulating osteogenesis	^269^
*Alk2*↑	↑p-S6 and mTORC1	severe craniofacial abnormalities	^195^
*Alk2*↑	↓autophagy	adopting a chondrogenic fate	^193^
*Bmpr1a*↓	↑Wnt (SOST, Dkk1)	bone mass ↑	^242,270^
*β-catenin*↓	↓responsiveness to BMP2	altering osteoblast differentiation	^267^
*β-catenin and Runx2*→	↑BMP9-induced	regulating osteogenesis	^268^
*BMP2*→	↑ cAMP-PKA/CREB/CRE	regulating osteogenesis	^274^
*Bmpr1a*↓	↑PKA signals enrichment	chondrocyte-to-osteoblast change	^204^
*Wnt*→	TGF-β type I receptor ↑	Runx2 ↑ osteoblast maturation ↑	^99^
*FGF2/9*→*FGF/FgfR*	↑ BMP2 and TGF-β1	↑osteogenic expression	^277^
*FGF2*→	↑BMP2 or ↑BMP receptor	regulating osteogenesis	^281,288^
*Bmpr1a*↑	↑FGF signaling	premature suture fusion	^202^
*FGFR3*→	↑degradation of Bmpr1a	skeletal dysplasia	^287^
*Notch signaling*↑	↑BMP-induced ALP	↑calcified nodules	^29136^
*BMP9*→	↑Hes1 level+ Smad signaling	regulating osteogenesis	^292^
*Shh (Gli2)→*	↑BMP2 promoter activity	regulating osteogenesis	^303^
*Ihh*→	↑BMP-induced osteogenesis	regulating osteogenesis	^306^
*Ihh and BMP*→	↑ALP, Ihh expression	regulating osteogenesis	^307^
*BMP6*↑	↑IGF1 activity	regulating osteoinductive effects	^309^
*IGF1*→	↑BMP2 expression	regulating osteogenesis	^316^
*IGF2*→	↑BMPR-Smad activity	BMP9-induced bone formation	^317^
*Atg7 siRNA*→	↓BMP2 activity	regulating osteogenesis	^319^
*Atg5*↓	↓BMP9 activity	regulating osteogenesis	^320^
*Bmpr1a*↓	↓Bmpr1a-mTOR/autophagy	osteoporosis pathogenesis	^321^
*BMP2*→	*↑*TGF-β3 activity	regulating chondrogenesis	^333^

↓decrease; ↑ increase; → stimulate; × block

During craniofacial development, increased Alk2 signaling in chondrocytes leads to severe craniofacial abnormalities and is associated with elevated S6 kinase phosphorylation and activation of mTORC1 (mammalian target of rapamycin complex 1).^194^ This suggests that Smad-dependent BMP signaling positively regulates the mTOR pathway, contributing to excessive nasal cartilage formation. However, it remains unclear whether BMP-driven mTOR activation alters osteoblast energy metabolism. Additionally, Alk2 activation inhibits autophagy in cranial neural crest cells by activating mTORC1, preventing autophagic degradation of β-catenin,^192^ and promoting chondrogenic lineage commitment in CNCs.

### Interplay between BMP and PKA/CREB signaling in osteoblasts and bone

Protein kinase A (PKA), a cAMP-dependent protein kinase, is a critical regulatory enzyme whose activity is tightly regulated by intracellular cAMP levels. In skeletal cells, cooperative interactions between CREB and BMP-regulated Smads are essential for bone formation.^267^ BMP2 signaling activates the PKA pathway to promote osteogenesis,^268^ a process that requires downregulation of protein kinase inhibitor gamma (PKIγ) to enable PKA activation. BMP signaling maintains balanced PKA activity by suppressing the adenylate cyclases Adcy5 and Adcy8.^269^

At the transcriptional level, CREB and Smad proteins form complexes that co-bind to BMP-responsive elements, thereby enhancing *Runx2* expression and osteogenic gene activation.^270^ *Bmpr1a* deficiency triggers a switch from chondrogenic to osteogenic, accompanied by the upregulation of PKA pathway genes.^203^ The BMP–PKA/CREB axis represents a druggable node that enhances bone formation without the need for high-dose BMP administration. Further studies are required to clarify its role in age-related bone loss and mechanical loading responses.

### Interplay between BMP and FGF signaling in osteoblasts and bone

The synergistic interplay between FGF and BMP signaling is crucial for bone formation and repair. FGF2 and FGF9 upregulate the expression of *BMP2* and *TGF-β1* in calvarial osteoblasts, with endogenous FGF/FGFR signaling acting as a positive upstream regulator of BMP2.^271^ Combined treatment with FGF2 and BMP2 enhances osteogenic capacity by increasing the expression of osteogenic markers (*osteocalcin*, *collagen type*-*I*, *Runx2*, *Osterix*, and *osteopontin*).^272^ The efficacy of BMP2 depends on FGF2; in *FGF2* mutant mice, BMP2-induced bone formation, ALP activity, and Runx2 nuclear accumulation are impaired.^273^ Runx2 is required to mediate *BMP2* expression in response to FGF stimulation during cranial bone development.^274,275^

Therapeutically, the combination of low-dose BMP7 and FGF2 optimizes bone regeneration.^276^ FGF2 and BMP2 exhibit synergistic effects during fracture repair, with FGF2 playing a more critical role in the early healing stages and BMP2 promoting mineralization in the later phases.^277^ The controlled release of FGF2 and BMP2 in the tissue-engineered periosteum significantly improves bone repair outcomes.^278,279^ Endogenous FGF2 levels influence the dosage required to amplify BMP2 activity, particularly in aged mice,^280^ triggering the question of why aging necessitates higher FGF2 levels to activate BMP signaling.

At the receptor level, FGFR3 facilitates the degradation of Bmpr1a, contributing to the pathogenesis of FGFR3-related skeletal dysplasia.^281^ The mechanism underlying the selective degradation of Bmpr1a, but not of other BMP receptors, by FGFR3 remains unclear. Low-dose FGF2 upregulates BMP receptor expression in progenitor cells^282^ (Fig. 3, Table 5). Intensified Bmpr1a signaling in cranial neural crest cells, but not in osteoblasts, drives premature suture fusion in mice, a process mediated by FGF signaling activation.^201^ This integrated framework highlights how FGF signaling primes BMP responsiveness across developmental, reparative, and aging contexts, thereby offering strategies for enhancing bone regeneration.

### Interplay between BMP and Notch signaling in osteoblasts and bone

The expression and function of Notch receptors (Notch1–4) in the skeleton are highly context-dependent and influenced by temporal and spatial expression patterns.^283^ Notch signaling activation enhances BMP-induced ALP activity and promotes the formation of calcified nodules in vitro,^284^ whereas Notch inhibition reduces ALP activity and decreases the promoter activity of BMP target genes.^285^ BMP9 induces periodic oscillations in *Hes1* mRNA levels in osteoblasts,^286^ facilitated by two Smad-binding sites in the mouse *Hes1* promoter.^286^ Key Notch pathway genes, such as *Hey1* and *Hes1*, are differentially regulated by BMP2 and TGF-β, serving as integration nodes for BMP-Smad and Notch signaling during osteoblast differentiation.^287^

BMP4–Notch cooperates to inhibit myogenic differentiation, redirecting progenitor cells toward the osteoblast lineage.^288^ CCN3, a matricellular protein, antagonizes BMP2 effects by modulating BMP and Notch pathways.^289^ Systemic inhibition of Notch signaling using dibenzoazepine (DBZ) impairs BMP2-induced calvarial bone healing in mice.^290^ However, BMP2 activates MAPK pathways (ERK, p38, and JNK) independently of Notch, regardless of the presence or absence of the Notch intracellular domain,^291^ suggesting that future studies are important for dissecting Notch–BMP crosstalk within distinct osteoblast subpopulations.

### Interplay between BMP, Hedgehog (Hh), and FGF signaling in osteoblast and bone

Hedgehog (Hh) signaling, mediated by Sonic (Shh), Indian (Ihh), and Desert (Dhh), plays a critical and context-dependent role in modulating BMP action during skeletal development. Unlike chondrogenesis, in which BMP signaling operates independently of Hh,^292–294^ BMP requires active Hh signaling to promote osteoblast differentiation.^292–294^ In the developing axial skeleton, sequential Shh and BMP signals are essential for specifying the chondrogenic fate of presomitic tissue.^295^ Shh cooperates with BMP2 to drive osteoblast differentiation^296^ by directly stimulating *BMP2* promoter activity through the transcription factor Gli2.^297^ Epigenetic upregulation of Hh pathway genes was observed during BMP7-induced osteoblast development.^298^ Conversely, BMP negatively regulates *Shh* transcription, creating a BMP–Shh negative feedback loop that restricts *Shh* expression during limb patterning^299^ (Fig. 3).

Ihh promotes osteoblast differentiation and inhibits chondrogenesis in mesenchymal progenitors.^300^ Ihh–BMP synergistically induces ALP activity and osteogenic marker expression.^301^ The disruption of *Smad1/5* impairs BMP–FGF–Ihh/PTHrP crosstalk, resulting in chondrodysplasia with abnormal endochondral growth.^229^ During craniofacial development, the coordinated activation of Hh, FGF, and Wnt/β-catenin signaling regulates suture morphogenesis and closure.^246^ In FOP, caused by mutations in ACVR1, cilia-mediated Hh signaling contributes to HO,^302^ suggesting that primary cilia serve as signaling hubs that integrate multiple pathways.

### Interplay between BMP and IGF signaling in osteoblasts and bone

IGFs and BMPs play synergistic roles in enhancing bone formation, both systemically and locally.^7,131^ Recombinant human BMP6 (rhBMP6) upregulates *IGF1* activity in primary human osteoblasts, accelerating mineralization.^303^ The combination of BMP6 and IGF1 enhances mineralization in bone tissue-engineered implants more effectively than BMP6 alone.^304^ During distraction osteogenesis, coordinated expression of *IGF1*, *TGF-β*, and *BMP4* suggests functional co-dependence among these pathways in bone repair.^305^

IGF1 and BMP7 enhance osteoblast differentiation^306^ and exhibit synergistic effects in promoting the chondrogenic differentiation of BMSC in rabbit knee injury models.^307^ Low doses of IGF1 maximally amplify BMP7-induced osteogenesis while minimizing the risk of hyperproliferation.^308^ Similarly, IGF1 and a BMP-derived P24 peptide act synergistically to promote the osteogenesis of BMSCs.^309^ IGF1 also upregulates *BMP2* expression during alveolar bone remodeling,^310^ and IGF2 enhances BMP9-induced ectopic bone formation in stem cell implantation assays.^311^

Mechanistically, IGF1 receptor (IGF1R) and BMP receptor (BMPR) signaling jointly activate BMP–Smad reporter activity and promote the nuclear translocation of Smad1/5/8.^311^ The BMP–IGF axis represents a powerful synergistic interaction in osteogenesis, underscoring the therapeutic potential of optimizing the dose–response curves for combined BMP–IGF treatments in bone regeneration.

### Interplay between BMP and autophagy in osteoblasts and bone

Autophagy, an evolutionarily conserved intracellular process for the selective degradation and recycling of cellular components,^312^ plays an essential role in BMP-induced osteogenic differentiation. BMP2/9-induced osteogenesis requires functional autophagy, and the inhibition of autophagy-related genes (e.g., *Atg7* or *Wnt16* via siRNA) blocks BMP2 effects.^313^ Chloroquine (an autophagy inhibitor) reduces the expression of *osteocalcin*, *osteopontin*, and *osterix*, whereas rapamycin (an autophagy inducer) enhances BMP-mediated mineralization.^313,314^ BMP9, among the most osteoinductive BMPs, upregulates key autophagy-related genes (e.g., *Atg5, LC3*) in MSCs,^314^ and *Atg5* knockdown impairs *Runx2* expression and late osteogenic markers.^314^

The conditional mutation of *Bmpr1a* in osteoblasts using *Col1a1-CreER* mitigates early bone loss in osteoporosis, but results in delayed bone maturation, reduced mineral apposition, and diminished bone formation rates,^315^ accompanied by compensatory dysregulation of mTOR-autophagy signaling.^315^ The activation of AMPK (an autophagy inducer) enhances osteoblast differentiation on titanium implant surfaces,^316^ suggesting a role for autophagy in fine-tuning BMP responses during implant healing. However, whether pharmacological induction of autophagy can overcome BMP resistance in aged bone remains unclear. Furthermore, a mechanistic interplay exists between mutant *Alk2-R206H* receptor signaling and the hypoxia-induced disruption of autophagic flux.^317^ Diminished autophagic flux increases the stability of the *Alk2-R206H* mutant,^317^ resulting in persistent signaling activation. This study identified aberrant autophagy as a central mechanism sustaining pathogenic Alk2-R206H signaling and revealed autophagic reactivation as a novel therapeutic strategy for FOP.

### Interplay between BMP and TGF-β signaling in osteoblasts and bone

The BMP and TGF-β pathways engage in context-dependent crosstalk that critically regulates skeletal development, homeostasis, and repair through both synergistic and antagonistic mechanisms. BMP9 promotes bone formation via JNK/Smad2/3 signaling,^318^ whereas periosteum-derived clonal cells (PDCs) activate both pathways upon ligand stimulation.^319^ Co-stimulation with BMP2 and TGF-β1 prolongs BMP-Smad1/5/8 signaling and downregulates the BMP antagonist Noggin.^320^ *TGF-β3* overexpression amplifies BMP2-driven osteogenesis,^321^ whereas GDF11 inhibits MSC osteogenesis via the Smad2/3-mediated repression of Runx2.^170^ Notably, the simultaneous inhibition of TGF-β and FGF signaling in the presence of BMP2 optimizes osteogenic differentiation in periostenum-derived MSCs (pASCs), with successful differentiation correlating with the early activation of p38 MAPK and Wnt/β-catenin pathways.^322^ BMP2 acts as a primary driver,^322^ whereas the TGF-β and FGF pathways act as modulators,^322^ suggesting that tailored modulation of the BMP, TGF-β, and FGF pathways will be essential for maximizing the regenerative potential of MSC-based therapies.^322^

In FOP, BMP9 mediates fibroproliferation through the aberrant activation of TGF-β signaling,^323^ with elevated phosphorylation of Smad2/3 and cellular proliferation in affected tissues. Systemic BMP9 neutralization or knockout mitigates disease flare-ups and HO in mouse models,^323^ revealing BMP9-induced TGF-β signaling as a trigger for FOP pathogenesis and a promising therapeutic target.

Advanced biomaterial systems leverage BMP-TGF-β synergy for enhanced regeneration. A TGF-β/chitosan and BMP2/silk fibroin hydrogel promotes the chondrogenesis of BMSCs in vivo and in vitro,^324^ supporting articular cartilage repair. Liposomes co-delivering BMP4 and TGF-β improve alveolar bone healing,^325^ and a bilayer system with affinity-bound TGF-β1 and BMP4 enhances endogenous cartilage regeneration.^326^ The BMP2/TGF-β3 combination in alginate culture exhibits superior chondrogenesis compared with TGF-β3 alone,^327^ and co-expressions of BMP2 and TGF-β3 intensify osteogenic differentiation in BMSCs.^321^ Under hypoxic conditions, BMP2–TGF-β1 co-delivery optimally induce chondrogenesis.^328,329^ Clinically, reduced TGF-β1 and BMP2 activities are observed in non-union patients^330^ (Fig. 3, Table 5), underscoring their potential as early indicators of fracture healing status.

BMP1 disrupts cell adhesion and stimulates TGF-β signaling in thrombospondin-1-rich microenvironments, influencing wound healing and tumor progression.^331^ The imprinted gene PEG10 is suppressed by TGF-β signaling and interferes with both TGF– and BMP–Smad pathways in chondrosarcoma, exerting dual roles in cell growth and invasion via AKT and p38 modulation,^332^ suggesting its potential as a therapeutic target. Further efforts should focus on optimizing combination therapies for non-union and degenerative joint diseases, as well as deciphering single-cell signaling dynamics within niches to advance precision regenerative medicine.

## TGF-β and BMP signaling in cartilage development and homeostasis

The TGF-β and BMP signaling pathways play pivotal roles in cartilage development and homeostasis, orchestrating the complex processes of endochondral ossification and chondrocyte differentiation. As an avascular and metabolically quiescent tissue, the cartilage relies on diffusive nutrient exchange and extracellular cues. During endochondral ossification, SSCs condense and commit to the chondrogenic lineage, establishing a transient cartilage template that guides subsequent bone formation. This template evolves through the highly coordinated phases of chondrocyte proliferation, maturation, and hypertrophic expansion. Terminally differentiated hypertrophic chondrocytes may then trans-differentiate or undergo dedifferentiation and re-differentiation into the osteogenic lineage, thereby contributing to bone formation. These dynamic processes are finetuned by crosstalk among multiple signaling pathways, including BMPs, TGF-βs, FGF, Indian Hedgehog (IHH), and parathyroid hormone-related protein (PTHrP), which collectively ensure spatiotemporal precision in skeletal morphogenesis and tissue homeostasis.

### BMP signaling in cartilage development and homeostasis

BMPs exhibit spatiotemporally regulated expression across the growth plate, metaphysis, epiphysis, and articular cartilage, where they orchestrate chondrocyte maturation and endochondral ossification. In the growth plate, BMPs (particularly BMP1–7) are highly expressed in hypertrophic and calcifying chondrocytes, osteoblasts, and vascular cells of the metaphysis.^333^ Similarly, articular chondrocytes in rats show a gradient of BMP expression from the superficial to the deep zones.^333^ During postnatal development in rats, BMP2/4 and BMP7 are strongly expressed in proliferating and maturing chondrocytes at 12 weeks,^334^ whereas their receptors (BMPR-IA, IB, and II) are co-expressed in these zones, but decrease upon hypertrophy. By 24 weeks,^334^ ligand expression declines, although receptor expression persists. Functional BMP signaling, assessed by phospho-Smad1/5/8, is most active in the proliferative and prehypertrophic zones,^335^ contrary to the ligand accumulation observed in hypertrophic regions, likely due to inhibitory Smad7 expression.^335^ Complementary gradients are observed in the articular cartilage; BMP agonists localize superficially, whereas antagonists are concentrated in deep zones,^335^ correlating with phospho-Smad activity. This spatiotemporal expression and signaling gradients suggest that BMP pathways critically regulate zone-specific chondrocyte differentiation in both the growth plate and articular cartilage, orchestrating postnatal endochondral ossification.

Furthermore, *BMP2, BMP7*, and *Bmpr1a* are upregulated in cartilage lesions and synovial tissues,^336^ highlighting their roles in repair. BMP signaling is essential for the initiation and differentiation of chondrocytes,^337^ with recombinant BMP2 (rhBMP2) regulating chondrocyte proliferation, differentiation, and matrix production in a maturation stage-dependent manner.^338^ Notably, BMP2 enhances glucose metabolism in primary chondrocytes by upregulating glucose transporter (*Glut1*) mRNA and protein levels,^339^ uncovering a novel metabolic link between BMP signaling and metabolic regulation in cartilage development.

Genetic studies have highlighted the essential role of BMP signaling in chondrogenesis. Double knockout of *Bmpr1a* and *Bmpr1b* in mice completely abolishes chondrocyte formation,^340^ whereas the chondrocyte-specific deletion of these receptors disrupts terminal chondrocyte differentiation.^341^ Bmpr1b-mediated signaling is indispensable for chondrocyte proliferation after chondrogenic commitment,^156^ and its function can be partially rescued by the constitutively active form of *Bmpr1a*, indicating that BMP signaling activation is necessary for both early chondrogenic differentiation and later hypertrophic maturation.^342^ Notably, the constitutive activation of *Bmpr1a*, but not *Bmpr1b*, leads to the expansion of Meckel’s cartilage,^343^ prevents its degeneration, and promotes endochondral ossification.

Downstream Smad1 and Smad5 are crucial effectors for maintaining chondrogenic differentiation; their ablation in chondrocytes causes severe chondrodysplasia.^229^ In contrast, Smad7 inhibits chondrocyte differentiation by suppressing BMP-activated p38 MAPK pathways.^344^ BMP signaling also regulates the self-renewal and quiescence of *Gli1*^*+*^ chondrogenic progenitors. *Bmpr1a* deletion disrupts *Gli1*^*+*^ progenitor quiescence and impairs columnar cartilage formation.^345^ Transcriptionally, BMPs orchestrate chondrocyte differentiation by modulating the expression of *Sox9*, a master regulator of chondrogenesis that maintains chondrocyte identity^346–348^ and suppresses Runx2/β-catenin-mediated hypertrophy.^349^ Postnatally, Sox9 prevents premature growth plate closure, and its ablation drives the trans-differentiation of chondrocytes into osteoprogenitor cells.^350^ Taken together, these findings highlight the indispensable role of BMP signaling in cartilage development, metabolism, and homeostasis. Future studies should explore whether metabolic interventions, such as targeting GLUT1, can enhance cartilage regeneration and whether age-related decline in BMP signaling contributes to disorders such as OA.

### TGF-β signaling in cartilage development and homeostasis

**Fig. 4:**
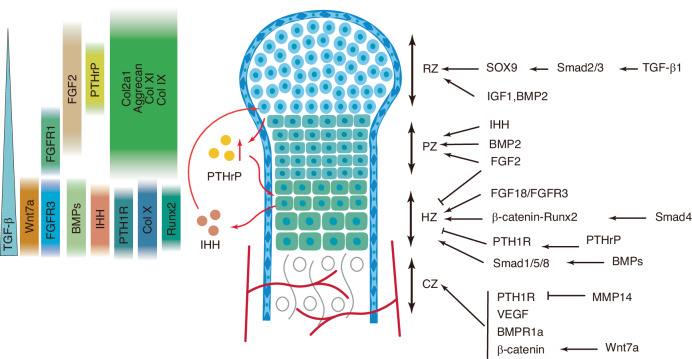
Spatiotemporal regulation of TGF-β/BMP signaling networks in cartilage. TGF-β/BMP signaling pathways display distinct spatial expression profiles and engage in intricate crosstalk with other major cascades, such as IGF, WNT/β-catenin, and FGF, across different growth plate zones (resting zone, RZ; proliferating zone, PZ; hypertrophic zone, HZ; calcified zone, CZ). These signaling networks collectively regulate chondrocyte proliferation, differentiation, and maturation during endochondral ossification. A central regulatory circuit, the Ihh-PTHrP feedback axis, critically controls the pace and spatial organization of chondrocyte differentiation. Both BMP and FGF pathways interact synergistically with the Ihh-PTHrP network within the growth plate, highlighting a multi-layered regulatory architecture that ensures precise coordination of skeletal growth

Chondrocytes produce all three TGF-β isoforms (TGF-β1, TGF-β2, and TGF-β3). TGF-β1 and TGF-β2 exhibit autoinduction, whereas TGF-β3 selectively upregulates *TGF-β1* expression without altering *TGF-β2* levels,^351^ thereby establishing context-dependent signaling gradients.^351^ Exogenous TGF-β1 reversibly inhibits terminal chondrocyte hypertrophy, thereby stabilizing the cartilage phenotype in a manner that depends on culture condition.^352^ It also regulates the synthesis of cartilage matrix proteins and metalloproteases.^352,353^ Notably, microtissues treated with TGF-β1 for 2 or 14 days have shown enhanced extracellular matrix production and chondrogenic gene expression compared with those treated with BMP2 or GDF5.^354^ TGF-β signaling further modulates articular chondrocyte proliferation and contributes to their activation during early OA.^355^ The systemic administration of recombinant human TGF-β2 in rats prevents the unloading-induced suppression of chondrocyte proliferation and epiphyseal growth plate expansion.^356^ The receptor Tgfbr2 is essential for regulating proteoglycan synthesis in both freshly isolated and cultured articular chondrocytes.^357^ During endochondral ossification, chondrocytes secrete a proteoglycan (PG)-rich extracellular matrix that inhibits cartilage maturation, including the expression of *Ihh* and *Col10a1*.^358^ The expression of truncated, kinase-defective *Tgfbr2* in mouse skeletal tissue promotes OA via accelerated terminal chondrocyte hypertrophy^359^ (Fig. 4).

Smad2 and Smad3 are critical mediators of TGF-β signaling in chondrocytes. The loss of *Smad2* and/or *Smad3* promotes the transition of resting chondrocytes into a highly proliferative columnar phase, whereas their presence maintains the quiescent state of resting chondrocytes in neonatal growth plates.^68^ Smad2 and Smad3 mediate TGF-β1-induced inhibition of chondrocyte maturation,^360^ with Smad3 specifically facilitating Sox9-dependent transcription during primary chondrogenesis.^361^ Smad4 regulates chondrocyte hypertrophy by upregulating *Runx2* expression and links Smad4-mediated Runx2 activation to skeletal development and chondrodysplasia.^79^ TGF-β/Smad3 signaling is essential for repressing articular chondrocyte differentiation,^72^ and perichondral Smad2 can partially compensate for Smad3 loss to restrain hypertrophy. Smad3 is specifically required for the TGF-β1-mediated control of chondrocyte proliferation.^362^ Beyond canonical Smad signaling, TGF-β also signals non-canonically, for instance, through p38 MAPK-mediated phosphorylation of Sox9 at serine 211,^93^ which enhances its stability. Additionally, TGF-βs strengthen gap junction communication via the Alk5–p-Smad3 axis,^363^ facilitating coordinated responses across chondrocyte populations.

### Integration of BMP, TGF-β, and key signaling pathways in cartilage biology

The Indian hedgehog/parathyroid hormone-related protein (Ihh/PTHrP) feedback loop constitutes a central regulatory axis governing growth plate development, coordinating chondrocyte proliferation, and hypertrophic differentiation.^364^ BMP signaling and its extracellular inhibitor Noggin, along with PTHrP, finely modulate Ihh activity in cartilage.^365^ The overexpression of *Ihh* upregulates *PTHrP* expression and delays hypertrophic transition.^364^ TGF-β exerts dual roles in endochondral ossification, regulating the tempo of hypertrophy both upstream of PTHrP and through a PTHrP-independent mechanism.^366^ TGF-β1 promotes chondrogenic gene expression through MAPK and Smad signaling pathways^367^ and regulates PTHrP production in chondrocytes, either downstream of or parallel to Ihh signaling.^368,369^ Smad4 is essential for maintaining normal *Ihh/PTHrP* expression^82^ (Fig. 4). Furthermore, the Tgfbr2–MCP-5 axis acts as a critical regulator of joint development and endochondral expansion,^54^ whereas Spry2 modulates signaling in both osteoblastic and chondrogenic lineages, playing a key modulatory role in endochondral bone formation.^370^

BMP signaling integrates with the Ihh/PTHrP loop,^371^ in which precisely calibrated BMP activity is essential for normal chondrocyte maturation and skeletal development.^372^ Functional studies indicate that Noggin-mediated BMP inhibition cannot counteract the effect of *Ihh* overexpression,^364^ and BMP2 fails to rescue the accelerated maturation caused by *Ihh* suppression.^373^ These findings suggest that BMP signaling delays hypertrophic differentiation through a mechanism parallel to and independent of the Ihh/PTHrP axis, raising an interesting question as to why the Ihh-PTHrP loop is strongly conserved, despite the existence of BMP-mediated regulation. Additionally, Prostaglandin E2 modulates chondrocyte development through direct engagement of the BMP/Smad cascade,^374^ whereas Shh and BMP signals form a positive feedback loop orchestrated by Sox9 and Nkx3.2, which potently activate chondrocyte differentiation across various cellular contexts.^375^

PTHrP primarily suppresses chondrocyte maturation through PKA/CREB signaling, whereas BMPs promote maturation via a Smad-dependent pathway. PTHrP-activated CREB signaling attenuates BMP2-induced expression of ColX and Ihh, but only weakly inhibits BMP-mediated Smad6 expression.^267^ The PG-rich extracellular matrix secreted by chondrocytes also restrains cartilage maturation and inhibits *Ihh* and *Col10a1* expression. TGF-β1 and BMP2 differentially stimulate PG synthesis and exhibit chondro-inductive properties.^376^ *Smad3* loss sensitizes chondrocytes to BMP2, leading to elevated *ColX* expression and enhanced Smad1/5/8 phosphorylation.^377^ During fracture repair, L-Sox5, Sox6, and Sox9 collaboratively drive and maintain chondrogenesis, a process augmented by BMP2 in an L-Sox5/Sox6/Sox9-dependent manner.^378^

FGF–BMP crosstalk is another critical regulatory layer. FGFR3 activation promotes Bmpr1a degradation, a key mechanism underlying FGFR3-related skeletal dysplasia.^281^ BMP2 partially rescues growth impairment in FGFR3 mutant models. Conversely, Dorsomorphin, an inhibitor of Bmpr1a, delays chondrogenic differentiation by suppressing BMP signaling and phenocopying the effects of FGF2 on chondrocytes. This highlights a finely tuned BMP–FGF axis, which is essential for cartilage development and homeostasis (Fig. 4).

## Multi-layered regulation in TGF-β/BMP signaling in bone

### Ligand antagonists of TGF-β/BMP signaling

The availability and activity of TGF-β and BMP ligands in the skeleton are tightly regulated by a network of extracellular antagonists, including Chordin, Follistatin, Noggin, Sclerostin, CTGF, and Gremlin (Grem), which modulate their signaling through distinct mechanisms.^379–383^ Notably, Noggin, Gremlin-1 (Grem1), and Gremlin-2 (Grem2) are transcriptionally induced in MSCs by TGF-β superfamily ligands via specific Smad pathways,^384^ forming negative feedback loops that fine-tune bone homeostasis.

Noggin, a prototypical BMP antagonist, plays a critical role in skeletogenesis, joint patterning, and vertebrate dorsal–ventral patterning.^381,385^ It shares structural homology with BMPs, including a conserved cystine knot topology,^386^ and inhibits BMP signaling by binding directly to the receptor-binding domain of BMP7,^387^ thereby blocking access to both type-I and -II BMP receptors.^387^ The overexpression of *Noggin* in skeletal cells leads to severe osteopenia and bone fragility due to BMP inhibition,^388,389^ whereas *Noggin* knockout mice exhibit cartilage hyperplasia and joint defects caused by uncontrolled BMP activity.^383,390^

**Fig. 5:**
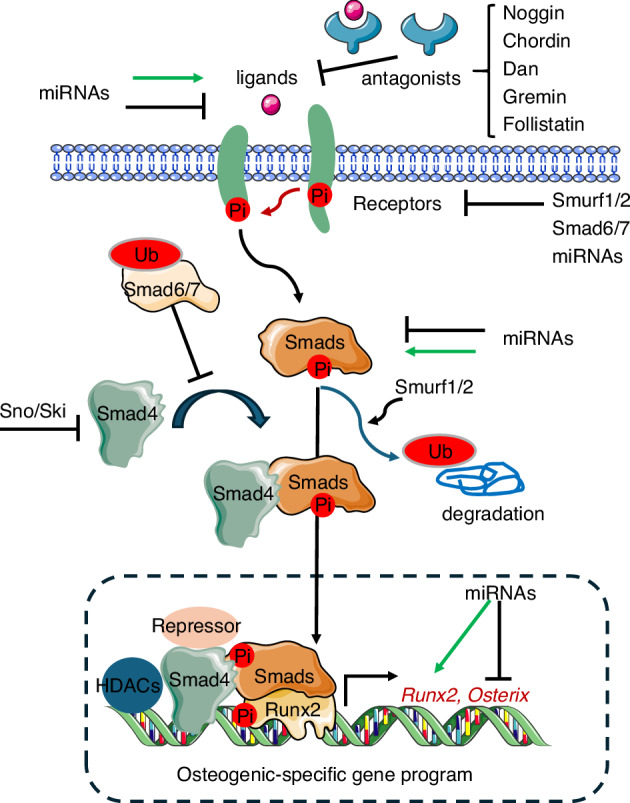
Multilayered negative regulation of TGF-β/BMP signaling in bone homeostasis. The TGF-β/BMP signaling pathway is precisely controlled through a multi-level inhibitory network. Extracellularly, soluble antagonists such as Noggin, Gremlin-1, and Chordin bind ligands and prevent receptor activation. Intracellularly, inhibitory Smads (I-Smads, Smad6/7) compete with receptor-activated Smads (R-Smads) for type I receptor binding and disrupt subsequent heterotrimeric complex formation with Smad4. The stability of signaling components is regulated by E3 ubiquitin ligases (e.g., Smurf1/2), which mediate K48-linked ubiquitination (Ub) and proteasomal degradation of activated R-Smads and receptors. Transcriptional control is achieved through corepressors (SnoN, Ski) that hinder Smad-DNA binding and recruit histone deacetylases (HDAC1/2) to repress osteogenic gene expression. Additional regulatory mechanisms include: (1) microRNAs (e.g., miR-21, miR-199a family) that post-transcriptionally modulate pathway components; (2) phosphatases (PPM1A, PP2A) that dephosphorylate receptors and Smads (phosphorylation, Pi)

Chordin delays chondrocyte maturation and negatively regulates endochondral ossification.^391^ Together with BMP ligands and intracellular effectors such as p-Smad, it forms an evolutionarily conserved self-organizing system.^392^ Chordin-related antagonists, including GDF3, exhibit structural features,^393^ such as a pre-oriented peptide loop that interacts with BMP2, and can function as either BMP inhibitors or context-dependent TGF-β ligands.^394^ CHL2, a chordin-like protein, adversely affects hyaline cartilage development and degeneration,^395^ emerging as a potential therapeutic target for OA (Fig. 5).

The DAN family of BMP antagonists, including Grem1 and Grem2, plays a pivotal role in modulating BMP activity within stem cell niches.^396^ Grem1 binds to heparan sulfate (HS) proteoglycans, forming localized BMP reservoirs in the extracellular matrix^382^ and exhibits pleiotropic functions across tissues and diseases.^382^ Unlike Noggin, DAN family members interact directly with type-I BMP receptor complexes.^397^ A notable feature is the ability of Grem2–GDF5 to form stable aggregate-like structures in vitro, which may preserve the signaling potential that is not shared by Follistatin or Noggin.^397^ How such aggregates maintain their contextual signaling competence remains an important question.

### Ubiquitin ligase-mediated degradation in TGF-β/BMP signaling

The ubiquitin–proteasome system plays a central role in regulating TGF-β/BMP signaling through targeted degradation of key pathway components, primarily orchestrated by Smad ubiquitination regulatory factors (Smurfs).^398^ Smurf1, a key negative regulator, mediates the degradation of BMP-activated Smad1/5 and the osteogenic transcription factor Runx2, thereby modulating bone mass.^399^ *Smurf1*-deficient mice exhibit age-dependent increases in bone mass,^400^ highlighting its role in bone remodeling. Structurally, the Smurf1 WW1 domain interacts with Smad1/5, enabling structure-based screening of small-molecule inhibitors.^401^ Mechanistically, Smurf1 interacts with its proline, glutamic acid, serine, and threonine motifs, which are recognized by p97, linking Smurf1 dysfunction to p97-related bone disorders.^402^ Similarly, Smurf2 forms complexes with Smad2 to target the transcriptional corepressor SnoN for degradation,^403^ and its overexpression in transgenic mice results in subchondral sclerosis and articular cartilage loss.^404^ Additionally, conserved cysteine residues in the BMP receptors facilitate Smurf1-mediated constitutive endocytosis, further regulating receptor turnover^405^ (Fig. 5).

Other ubiquitin ligases contribute to pathway fine-tuning: CHIP enhances Smad1/5 ubiquitination and degradation independent of chaperones,^406^ suppressing BMP signaling; the SCF (FBXL15) complex targets Smurf1 for degradation, playing a crucial role in embryonic development and adult bone formation^407^; and the fused (Fu) serine/threonine kinase collaborates with Smurf to ubiquitinate BMP receptors, controlling their proteolysis and turnover.^408^

Crosstalk between pathways further refines this regulation. TGF-β signaling upregulates *Smurf1* expression through the MAPK–ERK pathway,^409^ creating a feed-forward loop that degrades osteogenic proteins. Conversely, LMCD1 stabilizes Runx2 and Smad1 by preventing Smurf1-mediated ubiquitination,^410^ thereby promoting BMP signaling in osteoblasts.

Inhibitory Smad7 acts as a signaling node by recruiting Smurf2 to TGFβr1 for degradation.^411,412^ The overexpression of *Smad7* disrupts mesenchymal condensation and alters *Sox9* expression dynamics,^344^ impairing cartilage formation. Smurf1 also translocates Smad6 from the nucleus to the cytoplasm,^413–415^ and *Smad6/Smurf1* double-transgenic mice exhibit delayed endochondral ossification.^416^ Additionally, Id proteins are regulators of basic helix–loop–helix transcription factors, and *Id2* deficiency upregulates *Smad7* expression in chondrocytes,^417^ leading to dysmorphogenetic cranial base synchondroses. This multi-layered ubiquitin-dependent regulation ensures the precise control of TGF-β/BMP signaling outputs during skeletal development, homeostasis, and disease.

### Transcriptional repressors in regulating TGF-β/BMP signaling

The transcriptional corepressors Ski and SnoN play pivotal roles in modulating TGF-β signaling by exerting inhibitory effects within the nucleus.^418^ These repressors suppress target gene expression by disrupting the formation of functional Smad-DNA complexes.^418^ Mechanistically, Ski and SnoN bind to the MH2 domain of Smad4, a region that normally interacts with phosphorylated R-Smads (Smad2/3),^419^ thereby preventing the assembly of active Smad2/3-Smad4 complexes (Fig. 5). These repressors further prevent transcription through multiple mechanisms, including direct competition with the coactivator p300/CBP for binding surfaces (CH1/KIX domain of p300), induction of conformational changes in Smad4, and recruitment of histone deacetylases (HDAC) to promote chromatin compaction.^420^ By blocking coactivator recruitment and enhancing chromatin repression, Ski and SnoN effectively dampen TGF-β-mediated gene expression, highlighting their critical role in modulating the balance of BMP/TGF-β signaling during skeletal development and homeostasis.

### miRNA-mediated post-transcriptional regulation of TGF-β/BMP signaling

MicroRNAs (miRNAs), small non-coding RNAs of 19–22 nucleotides in length,^12^ play a pivotal role in post-transcriptional gene regulation and have emerged as key modulators of TGF-β/BMP signaling in skeletal biology.^421^ Hub miRNAs,^422^ including hsa-miR-27a/b-3p, hsa-miR-128-3p, hsa-miR-1-3p, hsa-miR-98-5p, and hsa-miR-130b-3p,^422^ coregulate both osteogenic and adipogenic differentiation factors, highlighting their dual roles in MSC fate determination. For instance, miR-10b suppresses Smad2 and partially inhibits TGF-β signaling,^423^ balancing osteogenic and adipogenic differentiation in human adipose-derived stem cells. During the BMP2-induced osteogenic differentiation of BMSCs, the downregulation of *miR-31, miR-106a*, and *miR-148a* further underscores the dynamic interplay between miRNAs and BMP signaling.^424^ Conversely, miR-26a inhibits BMP signaling by targeting Smad1, thereby impairing osteogenic differentiation in adipose-derived stem cells^425^ (Fig. 5).

Multiple miRNAs directly target the core components of the BMP pathway to form intricate regulatory circuits. miR-302 destabilizes *BMPRII* transcripts, creating a negative feedback loop with BMP4 to fine-tune BMP signaling.^426^ miR-148b enhances BMP2-induced osteogenesis by targeting the BMP antagonist, Noggin.^427^ miR-494 inhibits both Runx2 and BMPRII, which are key elements of the BMPR–Smad–Runx2 axis,^428^ whereas miR-106 isoforms suppress osteoblastic differentiation by targeting BMP2 and Smad5.^429^

miRNAs also regulate BMP/TGF-β signaling through downstream effectors. miR-195-5p activates the BMP2/Smad/Akt/Runx2 pathway by suppressing *Smurf1* expression.^430^ miR-421 targets Smad5, Id2, and Oct4,^431^ thereby modulating both BMP signaling and pluripotency. miR-296-5p directly targets *TGF-β1* mRNA, mitigating its inhibitory effects on chondrocyte survival and cartilage degradation via the CTGF/p38MAPK pathway.^432^

Clinically, the serum levels of miR-142 and BMP2 correlate with patient recovery and the RANKL/OPG ratio,^433^ suggesting their potential as biomarkers for bone healing. Additionally, TGF-β1-induced miR-191, regulated by Smad2 and BMI1 (Polycomb protein bmi-1), negatively impacts bone formation and regeneration,^434^ rendering miR-191 inhibition a promising therapeutic strategy for bone repair.

### Epigenetic regulation of TGF-β/BMP signaling

Epigenetic mechanisms, including histone modification, DNA methylation, and non-coding RNA regulation, play central roles in controlling gene expression and cellular functions during skeletal development and homeostasis. Histone deacetylases (HDACs) are key regulators of this process. HDAC1 deacetylates β-catenin to promote its degradation, thereby influencing chondrogenesis.^435^ HDAC4 suppresses chondrocyte hypertrophy while enhancing TGF-β1-induced chondrogenesis,^436^ and both HDAC4 and HDAC5 deacetylate Runx2, increasing its susceptibility to Smurf-mediated degradation.^437^ Smad7, a critical regulator of TGF-β signaling, is modulated by HDAC1-mediated deacetylation,^438^ which promotes its ubiquitination and degradation. Conversely, the acetylation of Smad7, facilitated by the ubiquitin ligase Smurf1, stabilizes the protein,^439^ demonstrating the dynamic interplay between acetylation, deacetylation, and ubiquitination in TGF-β signaling regulation. DNA methylation also significantly influences skeletal biology^440^ by regulating genes such as ALP during the osteoblast–osteocyte transition and modulating *SOST* expression in articular chondrocytes via altered Smad1/5/8 binding affinity to its promoter^441^ (Fig. 5).

Histone methyltransferases (e.g., DOT1L, EZH2, and G9a) and demethylases (e.g., KDM4B, KDM6A, KDM6B, and LSD1) critically regulate cartilage and bone development by modifying histone marks to influence transcription factors such as Runx2, NFAT1, and SOX9.^442^ ERK signaling enhances Runx2 stability and activity by increasing p300-mediated acetylation.^443^ NFAT1 acts as a transcriptional corepressor of the osteocalcin promoter, potentially via HDAC-dependent mechanisms.^444^ The CTR9–H3K27me3–BMP2 axis represents a novel epigenetic mechanism governing osteochondral lineage specification,^445^ with CTR9 preferentially promoting the osteoblast and chondrocyte differentiation of human MSCs. G9a binds to Runx2-targeted promoters and its mutation in cranial neural crest cells causes severe cranial bone hypomineralization.^446^ SETDB1-mediated H3K9m3 suppresses *OTX2* expression in osteoblasts.^447^

Epigenetic regulation also influences MSC migration, with TGF-β1-induced migration partially mediated by H3K27me3 and significantly impacted by the demethylase KDM6B.^448^ Methylation of the arginine residue R81 in Smad6 enhances its inhibition of Smad1–Smad4 complex formation and nuclear translocation.^449^ CBFA2T2 inhibits EHMT1-mediated histone methylation at the *Runx2* promoter,^450^ enabling BMP2-induced osteogenic differentiation. The zinc-finger transcription factor GATA4 maintains open chromatin at *Runx2* regulatory regions to facilitate bone mineralization.^451^ PRMT1 modulates BMP signaling and calvarial bone development through SMAD methylation, and its inhibition reduces BMP activity and H4R3me2a deposition.^452^

Notably, BMP signaling-dependent lactate production drives histone lactation, altering the expression of key genes such as *Pdgfra*^193^ and regulating CNC behavior in vitro and in vivo. However, the lactation landscape of chondrocyte and osteoblast genomes remains unclear. These findings collectively underscore the complexity and multi-layered nature of epigenetic regulation within TGF-β/BMP signaling in skeletal biology.

## TGF-β/BMP signaling in skeletal dysplasia and associated disorders

### TGF-β/BMP signaling in skeletal dysplasia

Craniosynostosis is a congenital anomaly characterized by the premature fusion of one or more cranial sutures, leading to abnormal craniofacial morphology. In mice, constitutively active *Bmpr1a* (caBmpr1a) in CNC cells is sufficient to drive premature fusion of the anterior frontal suture.^453^ In humans, congenital lambdoid craniosynostosis (CS), which involves the parietal–occipital suture, has been linked to pathogenic variants of BMP pathway genes, including *ACVRL1* and *ACVR2A*^454^ (Table 6).

**Table 6:** Genetic mutations in TGF-β/BMP signaling pathways and associated skeletal diseases

Genetic basis	Diseases	Mechanistic insight	Therapeutics	Refererence
*Bmpr1a mutations (caBmpr1a)*	Craniosynostosis	Augmented BMP signaling; premature fusion of the anterior frontal suture	BMP antagonist-loaded biomaterials	^458^
*ACVRL1/ACVR2A variants*	Congenital Lambdoid Craniosynostosis	Augmented BMP signaling; early fusion of the cranial suture between the parietal and occipital bones		^459^
ROR2 mutations	Robinow syndrome	Reduced p-Smad1/5/8 → impaired chondrogenesis	BMP2 supplementation trials	^483^
*ALK2-R206H*	Fibrodysplasia Ossificans Progressive (FOP)	GS domain hyperphosphorylation; BMPRII-enhanced mutant ALK signaling. progressive and extensive postnatal ossification of soft tissues	BLU-782 (ALK inhibitor); anti-ACVR1 antibody；anti-activin A monoclonal antibody；mTOR inhibitor	^460–463,472^
*Bmpr1b*	Brachydactyly Type A2	impaired phalanx joints formation; middle phalanx-specific defects	-	^473,474^
*GDF5 (N445K/T)*			-	^473,475,476^
*BMP2 regulatory duplications*			-	^477,478^
*Noggin mutations*	Brachydactyly Type B (BDB)	Unchecked BMP signaling; terminal digit loss	-	^481^
*TGF-β1 mutations*	Camurati-Engelmann disease	Vascular overgrowth; bone pain, fractures, and dysplasia	Targeted *TGFbr1 siRNA delivery*	^485,486,488^
*TGF-β signaling*	Osteogenesis Imperfecta	*Enhanced TGF-β signaling; high bone turnover*, bone fragility	*TGF-β neutralizing antibodies*	^492^
*Tgfbr1/Tgfbr2 mutations*	Loeys–Dietz Syndrome	Aneurysms skeletal abnormalities	Losartan (off-label use)	^489^
*excessive BmprII*	Fragile X Syndrome	enhanced BMPRII signaling, an inherited form of autism and intellectual disability	Potential inhibitor targeting the BMPRII-LIMK1 pathway	^484^
*active TGF-β1*	Enthesopathy	a problem with bone, tendon, or ligament insertion	Potential inhibitor	^487^
*FBN1 mutations*	Marfan Syndrome	TGF-β hyperactivity, long bone overgrowth, scoliosis, joint hypermobility	Potential inhibitor	^490,491^
*BmprII mutations*	Primary Pulmonary Hypertension (PPH)	obstruction of pre-capillary pulmonary arteries	BMP9 analogs in trials	^496^
*BMP15 defects*	Female Infertility	ovarian follicles, a common cause of female infertility	re-BMP15 supplementation	^493^
*BMP9*	Pulmonary Hepatopulmonary Syndrome	decreased oxygenation	-	^494^
*SMAD9 mutation*	osteoanabolic target for osteoporosis	a novel high bone mass gene	-	^231^

FOP is a rare genetic disorder caused primarily by gain-of-function mutations in ACVR1 (encoding ALK2), most commonly the p.Arg206His variant.^455–457^ This mutation confers constitutive BMP pathway activation and aberrant responsiveness to activins via impaired receptor autoinhibition,^185,458^ leading to progressive HO in soft tissues.^189,459^ Palovarotene (Sohonos™), the first FDA-approved treatment for FOP (2023), acts as a RARγ agonist that induces proteasomal degradation of Smad1/5/8/9, thereby attenuating BMP signaling.^460^ Its use is limited to specific age and sex groups because of the side effects observed in trials. In addition to palovarotene, several other mechanism-based therapeutics are currently under investigation. Small-molecule Alk2 inhibitors, including zilurgisertib (INCB000928) and fidrisertib (IPN60130), target receptor kinase activity,^461,462^ whereas saracatinib, a multi-kinase inhibitor, has exhibited efficacy in blocking HO in preclinical models.^462^ KER-047 inhibits activin A signaling by selectively targeting ALK4/5 receptors.^463^ Antibody-based strategies include anti-ACVR1 antibodies^464^ and the anti-activin A monoclonal antibody garetosmab (REGN2477), which reduces HO in murine models and may be most effective early in the disease.^458^ Additionally, the inhibition of the mTOR pathway has emerged as a promising adjunct strategy. Rapamycin suppresses the inflammatory and hypoxic potentiation of BMP signaling and has exhibited beneficial effects in patients,^465,466^ whereas BYL719, another mTOR inhibitor, prevents HO in cellular and animal models, supporting its potential for clinical evaluation^467^ (Table 6). These targeted agents reflect an increasing mechanistic understanding of FOP and offer complementary approaches for modulating dysregulated signaling pathways.

Brachydactyly Type A2 (BDA2), an autosomal dominant disorder characterized by malformations of the middle phalanges, is associated with mutations in *Bmpr1b*,^468,469^ *GDF5*,^468,470,471^ and *BMP2*.^472,473^ Specific mutations in *GDF5* (e.g., N445K/T) are also associated with synostosis syndrome,^474^ whereas duplications in the regulatory elements of BMP2^472,475^ contribute to BDA2 pathogenesis.^473^ In contrast, brachydactyly type-B (BDB), which is marked by terminal digit deficiencies, arises from point mutations in Noggin^476^ (Table 6).

Progressive pseudorheumatoid dysplasia, an autosomal-recessive skeletal disorder, involves the dysregulation of both BMP and Wnt/β-catenin signaling pathways.^477^ Robinow syndrome (RS), caused by loss-of-function mutations in *ROR2*, is characterized by reduced phosphorylation of Smad1/5/8.^478^ Fragile X syndrome (FXS), which results from FMR1 silencing, exhibits enhanced BMPRII signaling, suggesting that the BMPRII-LIMK1 axis is a therapeutic target for FXS and related autism spectrum disorders.^479^

Camurati–Engelmann disease (CED) is driven by activating mutations in *TGF-β1*,^480^ leading to diaphyseal osteosclerosis, thickened long bones, bone pain, and muscle weakness.^481^ Excessive TGF-β1 activity induces enthesopathy,^482^ characterized by vascular hyperplasia, bone degeneration, and fibrocartilage calcification. Targeted delivery of Tgfbr1 inhibitors has therapeutic potential, although systemic administration is limited by off-target effect.^483^ Loeys–Dietz syndrome, caused by mutations in *Tgfbr1* or *Tgfbr2*, exhibits hyperactive TGF-β signaling and presents with skeletal and vascular abnormalities, joint laxity, and increased fracture risk.^484^ Marfan syndrome results from mutations in fibrillin-1 (FBN1), encoding an extracellular protein that regulates TGF-β bioavailability,^485,486^ leading to secondary TGF-β hyperactivity and clinical features including long bone overgrowth, scoliosis, and joint hypermobility. Osteogenesis imperfecta (OI; brittle bone disease) is primarily caused by defects in COL1A1/2, but involves secondary TGF-β overactivity, contributing to bone fragility, growth deficiency, and blue sclerae. TGF-β inhibition in OI improves bone biomechanics and reduces elevated bone turnover, offering a promising therapeutic approach.^487^

Beyond skeletal disorders, aberrant TGF-β/BMP signaling contributes to a range of pathologies. Mutations in *BMP15* have been implicated in hypergonadotropic ovarian failure and female infertility.^488^ *BMP9* deficiency in rats causes hepatopulmonary syndrome, which is characterized by reduced oxygenation.^489^ Reduced endothelial BMP signaling in endothelial cells is linked to pulmonary arterial hypertension (PAH),^490^ and *BMPRII* mutations are associated with heritable primary pulmonary hypertension.^491^ These findings underscore the broad pathophysiological roles of TGF-β/BMP signaling and identify potential therapeutic targets. Future studies should focus on developing strategies to selectively block mutant signaling (e.g., Alk2) without impairing wild-type receptor function and on establishing predictive human disease models, including stem cell-based systems for drug screening. Notably, context-specific effects of TGF-β/BMP signaling must be considered; for example, BMPRII loss promotes PAH, whereas gain-of-function contributes to FXS, underscoring the need for tissue-specific therapeutic modulation.

### TGF-β/BMP signaling in osteoporosis

Low bone mass, a hallmark of osteoporosis (OP), significantly increases the risk of fragility fractures. The zinc-finger transcription factor GATA4 plays a critical role in multiple tissues, including the bone. Osteoblast-specific deletion of *GATA4* results in perinatal mortality and an osteoporotic phenotype characterized by reduced trabecular bone mass and diminished pSMAD1/5/8 signaling in the femoral trabecular region.^492^ MSCs from osteoporotic individuals exhibit impaired responsiveness to exogenous BMP2, indicating compromised BMP pathway activity.^493^ This aligns with the evidence that the β-catenin/BMP2/MAPK axis helps to maintain bone homeostasis and confers protection against diabetic osteoporosis.^494^

Notably, Smad9 has been identified as a novel high-bone-mass gene, representing a promising osteoanabolic target.^230^ In ovariectomized (OVX) rats, recombinant human BMP2 (rhBMP2) promotes spinal fusion and enhances early bone formation, indicating its therapeutic potential for improving spinal fusion outcomes in osteoporotic patients.^495^ Furthermore, osteoblastic Bmpr1a mediates hyperthyroidism-induced osteoporosis by augmenting osteoblast activity and osteoclast formation.^496^ The deletion of *Bmpr1a* in osteoprogenitors prevents hyperthyroidism-induced bone resorption and osteoporosis.^496^ *Bmpr1a* deficiency in early osteoporotic preosteoblasts dysregulates mTOR-autophagy signaling,^315^ leading to transiently enhanced proliferation, but ultimately impaired mineralization.

TGF-β superfamily signaling is significantly enriched in MSCs derived from individuals with primary osteoporosis, characterized by enhanced expression of the genes mediating Smad3-dependent TGF-β signaling and those encoding TGF-β antagonists.^384^ As a pivotal regulator of osteoblast differentiation, TGF-β plays a central role in coupling bone formation to resorption during remodeling.^497,498^ The constitutive activation of *Tgfβr1* induces the osteopenia phenotype,^50^ and TGF-β receptor inhibitors, currently in phase I/II clinical trials for cancer,^499^ may also serve as adjuvant therapies for osteoporosis. The inhibition of TGF-β increases bone volume and strength in a mouse model of osteogenesis imperfecta^487^ and trabecular bone mass.^500^ Conversely, osteoblast-specific overexpression of TGF-β2 results in an osteoporosis-like phenotype.^31^

Elevated serum TGF-β3 levels are associated with osteoporosis in women, suggesting their utility as early diagnostic biomarkers.^501^ Genetic studies further implicate TGF-β pathway components in disease susceptibility; specific SNP in TGF-β1 (rs1982073 C>T) and combinations of IL-10 (+1 927) variants^502^ correlate with increased fracture risk and ethnicity-specific predisposition to postmenopausal osteoporosis.^503^ Mechanistically, TGF-β intersects with BMP pathways in regulating MSC fate.^493^ TGF-β1 suppresses BMP2 expression through Tmeff1, thereby inhibiting BMP signaling and influencing MSC osteogenic differentiation.^504^ Notably, BMP9 attenuates microgravity-related disuse osteoporosis by modulating TGF-β and BMP signaling.^505^ Disuse osteoporosis, a subtype of osteoporosis caused by the absence of mechanical stress, shares striking pathological similarities with age-related osteoporosis. These findings highlight TGF-β inhibition as a promising therapeutic strategy for promoting bone formation, partly through potentiation of endogenous BMP signaling, avoiding the need for exogenous BMP administration and its associated risks, such as excessive bone resorption. Clinical translation, however, requires challenges to be overcome, including achieving bone-specific targeting of TGF-β inhibitors (e.g., via bisphosphonate conjugation) and defining safe regimens for short-term blockade versus chronic management of osteoporosis.

### TGF-β/BMP signaling in osteoarthritis

OA, a prevalent and debilitating joint disorder, does not require effective medical treatment due to our incomplete understanding of its pathophysiology. BMP2, which is minimally expressed in healthy cartilage, is significantly upregulated in OA tissue^506^ and frequently colocalizes with intracellular aggrecan, a feature absent from normal cartilage, but prominent in OA.^507^ BMP2 promotes matrix turnover even in IL-1-damaged cartilage, thereby facilitating natural cartilage repair.^506^ IL-1β upregulates *BMP2* expression in chondrocytes via the MEK/ERK/Sp1 pathway, while Noggin administration reduces BMP2/IL-1β-driven catabolism, mitigating OA progression and cartilage degradation.^508^ Genetic modification of *BMP2* in MSCs may enhance their therapeutic potential in OA-related cartilage repair.^509^

**Fig. 6:**
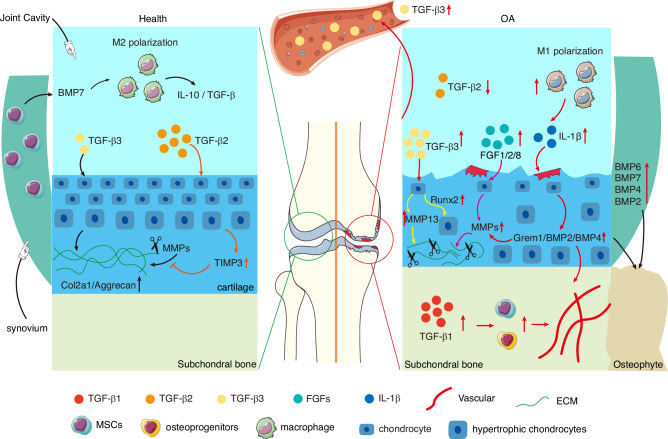
Dysregulation of TGF-β/BMP signaling in osteoarthritis pathogenesis. TGF-β/BMP signaling pathway exerts cell-type-specific and often opposing roles in osteoarthritis (OA) progression. In articular chondrocytes, aberrant activation of TGF-β (particularly TGF-β3 isoform) induces pathological hypertrophy and matrix degradation by upregulating MMPs (MMP-13, ADAMTS-5) while simultaneously inducing the BMP antagonist *Gremlin-1*, shifting the joint environment toward a catabolic state. In synovial fibroblasts, elevated BMP signaling enhances Smad1/5/8 phosphorylation, driving inflammation and osteophyte formation. TGF-β1 released from subchondral bone promotes aberrant angiogenesis and sensory nerve ingrowth via ALK1-Smad1/5 activation in endothelial cells, further accelerating OA progression. Cytokine crosstalk intensifies OA. IL-1β synergizes with FGFs to amplify TGF-β-mediated catabolism, while paradoxically increasing *BMP2/7* expression, forming a feed-forward loop that disrupts matrix homeostasis. Macrophage polarization also modulates OA pathogenesis, M1 macrophages secrete TGF-β-activated-kinase-1 (TAK1) to promote chondrocyte dedifferentiation, while M2 macrophages produce BMP6 to stimulate compensatory matrix synthesis

In patients with knee OA, *Grem1*, *BMP2*, and *BMP4* are co-expressed in the subchondral bone and deep-layer chondrocytes, positively correlating with cartilage degradation.^510^ *Grem1* overexpression accompanies prehypertrophic to hypertrophic differentiation in murine articular chondrocytes.^510^ Recombinant Grem1 induces *Mmp3* and *Mmp13* expression in chondrocytes and osteoblasts, whereas BMP4 upregulates osteoclastogenesis- (*Rankl, Ccl2*) and angiogenesis-related (*Angptl4*) genes^510^ (Fig. 6). Meanwhile, BMP7-loaded exosomes alleviate inflammation and cartilage damage in knee OA by promoting M2 macrophage polarization.^511^ Functionalized collagen–HA scaffolds co-delivering BMP7 and IL-10 mitigate early inflammation and foster a regenerative microenvironment during bone healing^512^ (Fig. 6).

Bmpr1a has emerged as a potential biomarker of OA progression in human knees with localized cartilage defects.^513^ Enhanced BMP signaling, marked by increased pSmad1/5/9, coincides with *Smurf1* depletion in OA cartilage, and BMP gain-of-function mutations alone are sufficient to induce OA in mice.^514^ Consequently, blocking local BMP signaling is a promising therapeutic strategy.^514^ Epigenetic regulation also plays a role; DNA methylation regulates *SOST* expression in OA via Smad1/5/8 binding.^441^ These findings suggest that BMP pathway modulation, either through inhibition or targeted delivery, has a viable therapeutic potential for OA,^515^ through the precise spatiotemporal dynamics of BMP signaling in OA pathogenesis requires further elucidation.

Dysregulated TGF-β signaling drives multiple pathological processes in advanced OA. Subchondral bone remodeling is closely linked to osteocyte-derived TGF-β signaling.^73^ Elevated active TGF-β1 levels in subchondral bone initiate degenerative changes, supporting TGF-β1 inhibition as a promising therapeutic strategy.^516^ In contrast, TGF-β3, which is abundant in OA joints, modulates chondrocyte metabolism by promoting mitochondrial fission through p-Smad3 and AMPK signaling^517^ (Fig. 6). Inflammation disrupts Smad2/3 signaling in chondrocytes through linker region (de)phosphorylation, offering a novel therapeutic target.^518^ Additionally, the TGF–β/TAK1–FoxO1 axis regulates articular cartilage autophagy and homeostasis, presenting a therapeutic target for OA-like joint disorders,^519^ while the local delivery of Cbfβ preserves TGF-β signaling in cartilage and may prevent OA progression.^520^ ECM biosynthesis-guided scaffolds loaded with TGF-β promote neocartilage regeneration,^521^ highlighting a translational strategy for structural cartilage repair.

Emerging translational studies highlight the complex and stage-specific roles of TGF in OA. A bone-targeting extracellular vesicle platform (BT-EV-G) has been found to inhibit pSmad2/3 and attenuate subchondral bone remodeling in a mouse OA model,^522^ whereas rAAV-mediated TGF-β gene therapy has been found to reduce perifocal OA progression and enhance osteochondral repair.^523^ Signaling modulation also influences cellular metabolism: TGF-β and BMP4 inhibitors alter mitochondrial activity in synovial fluid cells (SFCs), with BMP4 concentration directly influencing SFC activity independently of OA status.^524^ Furthermore, TGF-β suppresses IL-6 signaling in chondrocytes by downregulating IL-6R, mitigating pro-inflammatory effects, and maintaining cartilage homeostasis.^525^ TGF-β also induces nerve growth factor expression in chondrocytes via the ALK5–Smad2/3 axis, suggesting a non-inflammatory mechanism contributing to OA-related pain.^526^ Low-dose Alk5 inhibitors (e.g., SB505124) preserve Smad2/3 signaling while inhibiting *Runx2* expression and fibrosis in a subset of patients with OA.^527^ These findings underscore the multifaceted roles of TGF-β and BMP signaling in OA, highlighting isoform-specific effects (BMP2 vs. BMP7, and TGF-β1 vs. TGF-β3), stage-dependent strategies (early OA: anabolic; and late OA: catabolic blockade), and innovative delivery (EVs, gene therapy, and scaffolds), offering diverse therapeutic avenues for this complex disease. However, clinical translation faces challenges, including a lack of consensus biomarkers, particularly BMP/TGF-β ratios for OA staging, the lack of an available effective drug, and the need for spatially precise delivery to avoid systemic effects.

## TGF-β/BMP signaling in bone regeneration

BMPs are among the most potent osteoinductive factors identified. Currently, two BMP products, Infuse BMP2 (Medtronic) and OP-1 BMP7 (Stryker Biotech), are FDA-approved for clinical applications in treating long bone fractures and spinal fusion.^528^ These BMPs are delivered through a purified collagen-based scaffold implanted at the fracture site. BMP2 has demonstrated both clinical and economic benefits in acute open tibial fractures and economic advantages,^528^ whereas BMP7 achieves a 90% healing rate and 70% functional improvement in various joint fusion cases (ankle, subtalar, talonavicular, pubic, and sacroiliac).^529^ However, some limitations remain, including ectopic bone formation, inflammatory responses, and the short half-life of BMPs. BMP7 also shows promise for mitigating adynamic bone diseases associated with chronic kidney disease.^530,531^ When combined with a type-I collagen carrier, it exhibits a favorable safety profile in revision surgery and bone grafting.^532^ Recently, BMP9 has emerged as the most potent inducer of osteogenic differentiation in MSCs both in vitro and in vivo.^533^

An ideal BMP delivery system should provide spatiotemporally controlled release, often in combination with other factors, to maximize regenerative efficacy. For example, biodegradable nanospheres within collagen-like nanofibrous scaffolds enable the staged release of FGF2 and BMP7, optimizing bone growth through distinct kinetic profiles.^276^ Numerous BMP2-based strategies have been developed, including gene-loaded stereosomes,^534^ silk scaffolds functionalized with rhBMP2,^535–538^ and various biomaterial-based carriers.^539–545^ Cell-based strategies such as the implantation of BMP2-expressing stem cells also hold significant potential for personalized orthopedics and cranio-maxillofacial surgery.^546,547^ The secretome of BMP9-stimulated MSCs exhibits considerable osteogenic activity, suggesting a promising cell-free therapeutic avenue.^548^ Furthermore, the coordinated modulation of the BMP9-HIF-1α circuit can enhance bone repair,^549^ and the co-expression of *BMP2* and *TGF-β3* in BMSCs synergistically promotes osteogenesis.^550^ Interestingly, osteoclast activity attenuates BMP2-induced healing, suggesting that adjunct RANKL inhibition may improve the efficacy of BMP.^551^

Suture stem cells (e.g., *Gli1*^*+*^,^552^ *Prx1*^*+*^,^553^ and *Axin2*^*+*^^554^ populations) exhibit high regenerative potential,^552^ enabling critical-sized calvarial defect healing within 2–4 weeks.^555,556^ These cells represent promising therapeutic targets; for instance, BMP2-engineered suture stem cells significantly enhance cranial regeneration.^557^ The disruption of *Bmpr1a* impairs suture maintenance and repair via Hedgehog signaling,^558^ underscoring the role of BMP pathway modulation in amplifying endogenous regenerative capacity.

In tendon–bone integration, the use of TGF-β1 and BMP2 accelerates the osteogenic differentiation of ligament-derived stem cells.^559^ The dual release of BMP2 and TGF-β3 promotes functional enthesis regeneration,^560^ whereas TGF-β3 serves as an MSC-homing factor that enhances bone repair through direct and recruitment-mediated mechanisms.^561^ Silk-based matrices conjugated with TGF-β3 improve chondrogenic differentiation of BMSCs while suppressing hypertrophy.^562^ TGF-β1 represents a critical target in impaired healing contexts,^504^ and its combination with TGF-β2 may offer early prognostic value for bone healing outcomes.^37^ Despite these progresses, clinical translation faces challenges, such as BMP2-related complications (e.g., edema) and the biphasic (dose-dependent) effects of TGF-β signaling. Future advances will likely rely on precision delivery platforms, combinatorial approaches including CRISPR-activated endogenous BMP expression, and AI-designed release kinetics to optimize the spatiotemporal control of growth factor activity.

## Summary and perspective

The TGF-β and BMP signaling pathways serve as master regulators of osteoblast differentiation and bone formation, playing indispensable roles in skeletal development, homeostasis, and repair. Signaling occurs through both canonical (Smad-dependent) and non-canonical (e.g., p38 MAPK and PI3K/AKT) mechanisms, converging on master transcription factors such as Runx2 and Osterix to orchestrate lineage-specific gene expression programs. Beyond driving MSC commitment, TGF-β/BMP signaling pathways precisely modulate the equilibrium between bone formation and resorption, thereby maintaining skeletal integrity over a lifetime. Advances in conditional genetics, functional genomics, and systems biology have unveiled extensive crosstalk with Wnt/β-catenin, Hedgehog, Notch, autophagy, IGF, and FGF signaling, deepening our understanding of bone biology at the molecular level.

Clinically, dysregulated TGF-β/BMP signaling contributes to diverse disorders, spanning skeletal malformations, metabolic bone diseases, and malignancies. Gain-of-function mutations in ACVR1/Alk2 drive the progression of fibrodysplasia ossificans, whereas diminished BMP activity has been implicated in osteoporosis and compromised fracture healing. With aging populations projected to double by 2050, osteoporosis-related complications, particularly hip fractures, will impose an escalating socioeconomic burden, with annual healthcare expenditures exceeding $17 billion in the U.S. alone. This urgency underscores the need to elucidate the precise pathophysiological mechanisms governing skeletal remodeling in health and disease.

Therapeutically, recombinant BMP2 and BMP7 have been used in over one million clinical cases worldwide, revolutionizing the treatment of long bone non-union, spinal fusions, and complex fractures. However, their utility is limited by the need for high doses, substantial costs, and adverse effects, such as ectopic ossification and inflammation. Innovative strategies are emerging to overcome these challenges, including: (1) novel biologic agents (e.g., BMP9 analogs); (2) smart biomaterials for spatiotemporally controlled release; and (3) cost-effective prevention strategies for aging populations. Recent studies have highlighted the potential of BMPs beyond skeletal tissues, demonstrating their roles in renal repair, glucose regulation, and iron metabolism.

To fully harness TGF-β/BMP pathways for therapy, future investigations should prioritize the following key areas: (1) Mechanistic specificity: Using single-cell multi-omics and advanced imaging to decode context-dependent signaling outcomes. (2) Precision delivery: Developing tissue-targeted delivery systems (e.g., exosome-encapsulated BMPs) and gene-editing approaches (e.g., CRISPR-based ACVR1 correction in FOP). (3) Long-term safety and efficacy: Evaluating oncogenic risks from chronic TGF-β inhibition and BMP overactivation using longitudinal animal models and patient-derived organoids. (4) System-level integration: Applying AI-driven modeling to optimize combinatorial interventions and define therapeutic windows. Multidisciplinary collaboration across structural biology, computational modeling, and clinical trials will be essential for translating these insights into safe and effective precision therapies, not only for skeletal diseases but also for fibrotic, cardiovascular, and metabolic disorders.

## Data Availability

All the data are within the manuscript.
